# Comparative Assessment of Statistical and Thermodynamic Prediction Methods for Solvate Formation: A Case Study with Curcumin and Its Derivatives

**DOI:** 10.1021/acs.cgd.5c01343

**Published:** 2025-12-09

**Authors:** Julian Ticona-Chambi, Duane Choquesillo-Lazarte, Silvia Lucia Cuffini, Lourdes Infantes

**Affiliations:** † Instituto de Ciência e Tecnologia (ICT), 28105 Universidade Federal de São Paulo (UNIFESP), São José dos Campos 12231-280, Brazil; ‡ Laboratorio de Estudios Cristalográficos, IACT-CSIC, Avda. de las Palmeras 4, Armilla 18100, Spain; § Instituto de Química Física Blas Cabrera (IQF), 16379 Consejo Superior de Investigaciones Científicas (CSIC), Serrano 11,Madrid 28006, Spain

## Abstract

This study compares statistical and thermodynamic methodologies for predicting solvate formation using curcumin (CUR) and its derivatives demethoxycurcumin (DMC) and bisdemethoxycurcumin (BDMC) as models. We evaluated the performance of Statistical Frequency of Interaction for Multicomponent Prediction (SFIMP) and Conductor-like Screening Model for Realistic Solvents (COSMO-RS) methods to identify solvents likely to form solvates. A comprehensive crystallization screen yielded several new solvated and hydrated forms. Our results show that hydrogen bond propensity (HBP) performed best among individual predictors, while COSMO-RS combined with HBP yielded superior predictive accuracy overall. These insights aid rational design and screening of multicomponent solid forms in pharmaceutical development.

## Graphical abstract

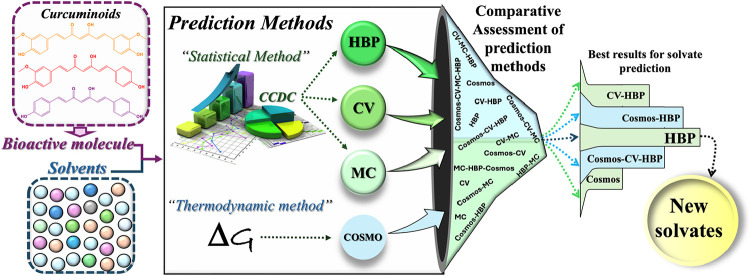

## 1 Introduction

Curcumin (CUR), a natural polyphenol derived from the rhizome of Curcuma longa, has attracted significant attention due to its wide-ranging biological properties, including anti-inflammatory, antioxidant, antimicrobial, and anticancer activities.^1–3^ Recent studies have demonstrated that curcuminoids possess promising potential not only as therapeutic agents but also as functional materials in fields such as photonics, sensors, and drug delivery systems.^4,5^ However, their application remains limited by poor aqueous solubility, low bioavailability, and physicochemical instability. Strategies such as nanoparticle formulation, cocrystallization, and solvate/hydrate formation have been explored to improve their pharmacokinetic and physicochemical properties.^6^

From a solid-state chemistry perspective, the formation of solvates and hydrates can dramatically affect a compound’s physical and chemical properties, including solubility, melting point, stability, and polymorphic behavior.^7–9^ In the case of curcuminoids, several solvate and hydrate forms have been reported, particularly for CUR and bisdemethoxycurcumin (BDMC), illustrating their capacity to engage in specific supramolecular interactions with a variety of solvents.^10–17^ Conversely, demethoxycurcumin (DMC) remains less explored in this context, with no detailed structural reports of its solvated forms to date.

Predicting the formation of solvates remains a challenge in crystal engineering, as it involves understanding the balance of intermolecular forces, conformational flexibility, and thermodynamic stability. Several computational tools have emerged to support rational solid form screening, including statistical models based on experimental data (e.g., Hydrogen Bond Propensity (HBP), Molecular Complementarity (MC), Coordination Values (CV)) and thermodynamic methods such as the COSMO-RS (Conductor-like Screening Model for Realistic Solvents) approach.^18–20^ Notably, Costa et al.^21^ demonstrated the application of these statistical tools in the prediction of cocrystal formation for the antiretroviral drug nevirapine, showcasing their utility in multicomponent solid form screening. While these approaches have shown promise in cocrystal design, their comparative performance in forecasting solvate formation remains underexplored, particularly for structurally related compounds such as curcuminoids.

This study presents a comprehensive investigation of solvate and hydrate formation in curcuminoids, focusing on curcumin (CUR), demethoxycurcumin (DMC), and bisdemethoxycurcumin (BDMC). Several new crystalline forms are described, including previously unreported solvates and hydrates, and notably, the first crystal structure of DMC. These solid forms provide valuable structural insights and enable a systematic evaluation of predictive methodologies. We assess the performance of statistical (Hydrogen Bond Propensity, Molecular Complementarity, Coordination Values) and thermodynamic (COSMO-RS) tools in ranking solvents based on their likelihood to form solvates. By comparing computational predictions with experimental outcomes, this work contributes to the development of more effective strategies for solvate screening in pharmaceutical and materials science applications.

## 2 Methodology

### 2.1 Crystallization Process

CUR, DMC, and BDMC (Sigma-Aldrich) were used as received. Analytical-grade solvents were employed throughout. CUR was screened with 41 solvents, while DMC and BDMC were limited to 16 solvents each due to material availability. Recrystallization was performed under varied temperature and pressure conditions to optimize single-crystal growth (Tables S1 and S2). Key solvates were successfully obtained via slow evaporation (from 50 °C to room temperature) for CUR-DOX and DMC-DOX, and via cooling crystallization (heating to 30 °C, rapid cooling in liquid nitrogen and slow evaporation at 4 °C) for BDMC-DOX, BDMC-WATER1, BDMC-WATER-2, all under ambient pressure. Suitable crystals for X-ray diffraction were carefully selected and preserved (e.g., in capillaries or under cryogenic conditions when necessary).

### 2.2 Single Crystal X-ray Diffraction and Crystal Structure Analyses

Prior to X-ray data collection, specific precautions were taken to prevent degradation or dissolution of the crystals. The CUR-DOX solvate was unstable at room temperature and darkened upon contact with inert oil. To preserve its integrity, the crystal was placed inside a glass capillary along with a small amount of DOX solvent. In the case of BDMC-DOX, the crystal exhibited rapid dissolution when handled at ambient conditions. Therefore, it was picked up at 4 °C in a cold room using a cryoloop and immediately flash-frozen in liquid nitrogen before mounting on the diffractometer.

Single-crystal X-ray diffraction (SCXRD) data for CUR-DOX, DMC-DOX, and BDMC-DOX were collected using a Bruker APEX II diffractometer equipped with a microsource Cu Kα radiation and a Photon 100 CCD detector. Measurements were performed at room temperature for CUR-DOX, DMC-DOX, and CUR-DMSO, and at 120 K for BDMC-DOX. Diffraction data for BDMC-WATER-1 and BDMC-WATER-2 were collected at 100 K using synchrotron radiation at the ALBA Synchrotron facility (BL13-XALOC beamline).

Diffraction data collected on the APEX II instrument were processed with the APEX software suite,^22^ whereas synchrotron data sets were processed using XDS.^23^ All structures were solved by intrinsic phasing using SHELXT and refined by full-matrix least-squares on F^2^ using SHELXL,^24^ both implemented within the Olex2–1.5 platform.^25^ Non-hydrogen atoms were refined anisotropically. Disordered solvent molecules (1,4-dioxane) were observed in CUR-DOX and BDMC-DOX and modeled using appropriate disorder restraints and constraints.

For BDMC-WATER-2, two possible crystallographic cells were identified [*P*2_1_/*c*: 20.255(5) Å, 7.2390(6) Å, 53.895(4) Å, β = 90.034(7)°, Volume = 7902(2) Å^3^; *P*2_1_/*n*: 20.254(4) Å, 7.2380(4) Å, 11.5070(10) Å, β = 110.522(8)°, Volume = 1579.9(4) Å^3^]. We retained the former because it accounts for all reflections in reciprocal space and yielded lower R-factors in the refinement (6.6 vs 10.2).

For postrefinement structural analysis, several tools implemented in Mercury 2024.1.0 were employed. These included Full Interaction Maps (FIM), intermolecular potential surface visualization, Hydrate and Solvate Analyzer, and structural overlay comparison. These tools were used to assess hydrogen bonding patterns, molecular packing, and conformational variability across the new solvates and hydrates of CUR, DMC, and BDMC.

### 2.3 Prediction Tools

Two classes of prediction models were evaluated: The Statistical Analysis of Frequency of Interaction for Multicomponent Prediction (SFIMP)^21^ and the thermodynamic Conductor-like Screening Model for Realistic Solvents (COSMO-RS).^18^ The general flowchart, illustrated in Figure 1, summarizes these methodologies. A data set of forty-eight polar and nonpolar solvent molecules were classified for each analysis (See Supporting Information Table S1). According to values obtain in each method, a ranking and consensus ranking were established using different criteria, as presented below.

**Figure 1:**
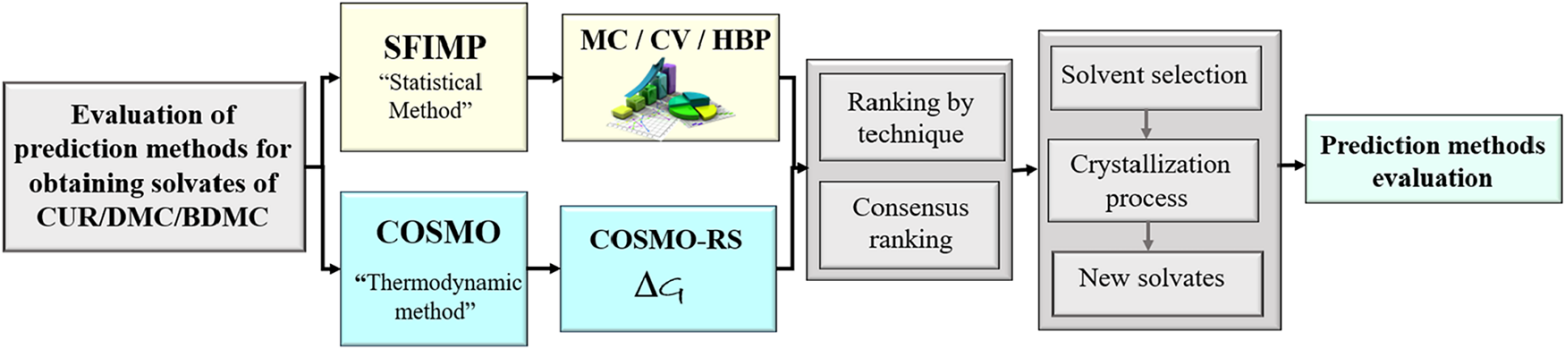
Flowchart summarizing the methodologies used to evaluate the probability of solvate formation.

#### 2.3.1 Statistical Approach—SFIMP

The Statistical Frequency of Interaction for Multicomponent Prediction (SFIMP) framework integrates three key descriptors: H-Bond Propensity (HBP),^26,27^ Molecular Complementarity (MC)^28^ and Coordination Values (CV),^29,30^ which were developed by the Cambridge Crystallographic Data Center (CCDC) and optimized by Costa et al., 2020.^21,31^ The procedure applied in each technique is described below

##### 2.3.1.1 HBP

The Hydrogen Bond Propensity (HBP) method estimates the likelihood of hydrogen bond formation between donor and acceptor functional groups based on statistical models derived from experimental crystal structures from the Cambridge Structural Database (CSD). The HBP tool evaluates each possible donor–acceptor interaction within a target system by assigning a propensity score (*P*). This score reflects the probability that a hydrogen bond is formed.

To assess the likelihood of multicomponent formation (e.g., target molecule with a solvent), the HBP* score is computed using the following formula (eq 1)­

1 $${\mathrm{HBP}}^{*}=\max\left(P_{\mathrm{target}‐\mathrm{molecule}}-P_{\mathrm{solvent}}\right)-\max\left(P_{\mathrm{target}‐\mathrm{molecule}}-P_{\mathrm{target}‐\mathrm{molecule}}\;\mathrm{or}\;P_{\mathrm{solvent}}-P_{\mathrm{solvent}}\right)$$

This equation compares the strongest heteromeric (target-solvent) interaction with the strongest homomeric (target–target or solvent–solvent) interaction. A positive HBP* score suggests that the heteromeric interaction is more favorable, indicating a higher probability of solvate formation. Conversely, negative values imply a preference for self-association, making solvate formation less likely.

Solvents are then ranked in descending order based on their HBP* values. This ranking reflects their potential to form stable hydrogen-bonded solvates with the target molecule.

##### 2.3.1.2 MC

Molecular Complementarity (MC) is a knowledge-based approach used to assess the geometric and electronic compatibility between two molecules, based on shape and polarity descriptors. It provides a measure of how well a pair of molecules might fit and interact in a solid-state environment, supporting the prediction of cocrystal or solvate formation. This method is particularly useful in multicomponent crystal engineering where shape complementarity and dipolar matching play crucial roles in molecular packing and stabilization.

Multiple conformations of the target compounds and solvents were generated using the Conformation Generator Tool available in Mercury. For each conformation, the molecular complementarity between the target molecule and each solvent was assessed using the Molecular Complementarity Screening Wizard implemented in Mercury. The resulting data table includes five key descriptors for both components: the medium (*M*), short (*S*), and long (*L*) molecular axes, the dipole moment, and the fraction of nitrogen and oxygen atoms (N/O). To compare the complementarity between pairs, these descriptors were normalized using the equation below (eq 2)­

2 $${\mathrm{MC}}_{N}=\frac{\Delta M/L}{0.31}+\frac{\Delta S}{3.23}+\frac{\Delta S/L}{0.275}+\frac{\Delta \mathrm{dipole}\;\mathrm{moment}}{5.94}+\frac{\Delta \mathrm{fraction\_N\_O}}{0.294}$$

MC Ranking Criteria: A lower MC_*N*_ value indicates greater molecular complementarity between the target compound and the solvent. Accordingly, solvents were ranked from lowest to highest MC_*N*_ values, with the lowest scores representing the most complementary solvent–target combinations.^21^

##### 2.3.1.3 CV

The Coordination Values (CV) method is a statistical approach that quantifies a molecule’s ability to act as a hydrogen bond donor or acceptor based on empirical data from crystal structures. It estimates the number of hydrogen bonds each functional group can form, accounting for both electronic and geometric factors that influence intermolecular interactions in the solid state. This method is particularly useful in predicting multicomponent formation by assessing whether the hydrogen bonding landscape of a coformer complements that of a target molecule.

Different conformations of each analyzed molecule (target molecules and solvents) were generated using the Conformation generator tool available in the Mercury software. These conformers were used to evaluate hydrogen bonding capacities by calculating the coordination values of each structure using custom Python scripts based on the CSD Python API. These CV scripts assign a donor coordination value (*D*_target‑molecule_, *D*_solvent_, *D*_multicomponent_) and an acceptor coordination value (*A*_target‑molecule_, *A*_solvent_, *A*_multicomponent_) to each molecule.

The donor and acceptor capacities represent the estimated number of hydrogen bonds each molecule can form as a donor or acceptor. The difference between these capacities was computed as an absolute value (|D–A|), which reflects the balance or imbalance of hydrogen bonding potential.

The degree of coordination mismatch—or “comfort”—in the multicomponent system (solvate) relative to the pure components was calculated using the following expression (eq 3). A worked example is provided in the worked-out example section of the Supporting Information.

3 $$\Delta \mathrm{CV}=\left(\left|({\left(D\text{−}A\right)}_{\mathrm{solvate}}\right|-\left|({\left(D\text{−}A\right)}_{\mathrm{targetmolecule}}\right|\right)+\left(\left|({\left(D\text{−}A\right)}_{\mathrm{solvate}}\right|-\left|({\left(D\text{−}A\right)}_{\mathrm{solvent}}\right|\right)$$

CV Ranking Criteria: Solvents were ranked based on increasing ΔCV. Lower values indicate a greater likelihood of favorable hydrogen bonding complementarity in the multicomponent form.^21^

#### 2.3.2 Thermodynamic Approach—COSMO-RS

The COSMO-RS (COnductor-like Screening MOdel for Real Solvents) approach is a quantum chemistry-based method that predicts the thermodynamic behavior of molecular interactions in solution or solid state. It combines electronic structure calculations with statistical thermodynamics to estimate the excess enthalpy (Hex) of mixing between molecular pairs. This descriptor reflects the strength of interaction between a target molecule and a solvent and can be used to assess the likelihood of solvate formation.

In this study, the COSMOQuick 1.9 software^32^ was employed to perform COSMO-RS calculations. SMILES representations of all target molecules (CUR, DMC, BDMC) and solvents were obtained from the PubChem database (See Supporting Information Table S1). These SMILES were used as input to compute the excess enthalpy of interaction between each target–solvent pair.

The COSMO-RS model operates by placing molecules in a virtual conductor environment and analyzing their surface polarization charge densities (σ-surfaces). Interaction energies are derived from the statistical thermodynamic treatment of surface interactions, which include contributions from hydrogen bonding, electrostatics, and van der Waals forces.

The excess enthalpy of formation (Hex) between each pair reflects the thermodynamic favorability of interaction: more negative Hex values indicate stronger interactions, suggesting a higher potential for solvate formation.

targetmolecule+solvent→solvate,⁣ΔHex<0

COSMO-RS Ranking Criteria: Solvents were ranked in ascending order of Hex values. Those with the most negative excess enthalpies were considered to have the highest likelihood of forming thermodynamically stable solvates with the target compound.^21^

#### 2.3.3 Combined Ranking and Evaluation

Since each predictive method captures different aspects of molecular behavior—such as hydrogen bonding propensity, electrostatic complementarity, or donor–acceptor balance—combined rankings were generated to assess their collective predictive power. These Consensus Rankings (CRs) integrate the strengths of multiple descriptors and allow evaluation of synergistic effects between statistical and thermodynamic models. A series of combinations were constructed by summing the rank positions of each solvent across different pairs or groups of methods. The combinations tested included: COSMO–CV, COSMO-MC, COSMO-HBP, CV-MC, CV-HBP, HBP-MC, COSMO–CV-MC; COSMO–CV-HBP, CV-MC-HBP, MC-HBP-COSMO, CV-MC-HBP-COSMO. For each combination, the consensus rank of a solvent was obtained by calculating the arithmetic sum of its rank positions in each individual method. Lower consensus values indicate better agreement across methods regarding that solvent’s ability to form a stable solvate.

To evaluate the performance of both individual and combined prediction methods, we compared their ability to correctly rank solvents that are known (from literature) or were found experimentally (see Section 2.2) to form solvates with CUR and BDMC. For each method, the positions of all confirmed solvating solvents in the ranked list were summed and then averaged (eq 4)­

4 $$\mathrm{mean}\;\mathrm{rank}=\frac{\sum \left(\mathrm{positions}\;\mathrm{of}\;\mathrm{confirmed}\;\mathrm{solvates}\right)}{\mathrm{number}\;\mathrm{of}\;\mathrm{solvates}}$$

A lower mean rank value implies that the method consistently ranked actual solvating solvents closer to the top of the list, thereby indicating higher predictive performance. This approach enables a direct comparison between the relative accuracies of individual and combined methodologies in identifying suitable solvents for solvate formation.

## 3 Results and Discussion

### 3.1 Structural and Supramolecular Features of Curcumin and Its Derivatives in the Solid State

Structurally, CUR consists of two aromatic rings connected by a conjugated keto–enol linker, containing both phenolic and enolic functional groups capable of engaging in various intermolecular interactions (Figure 2). Two naturally occurring derivatives, DMC and BDMC, share the same core skeleton but differ in the number of methoxy substituents on the aromatic rings. These subtle structural differences influence their chemical reactivity, biological efficacy, and solid-state behavior.^33,34^

**Figure 2:**
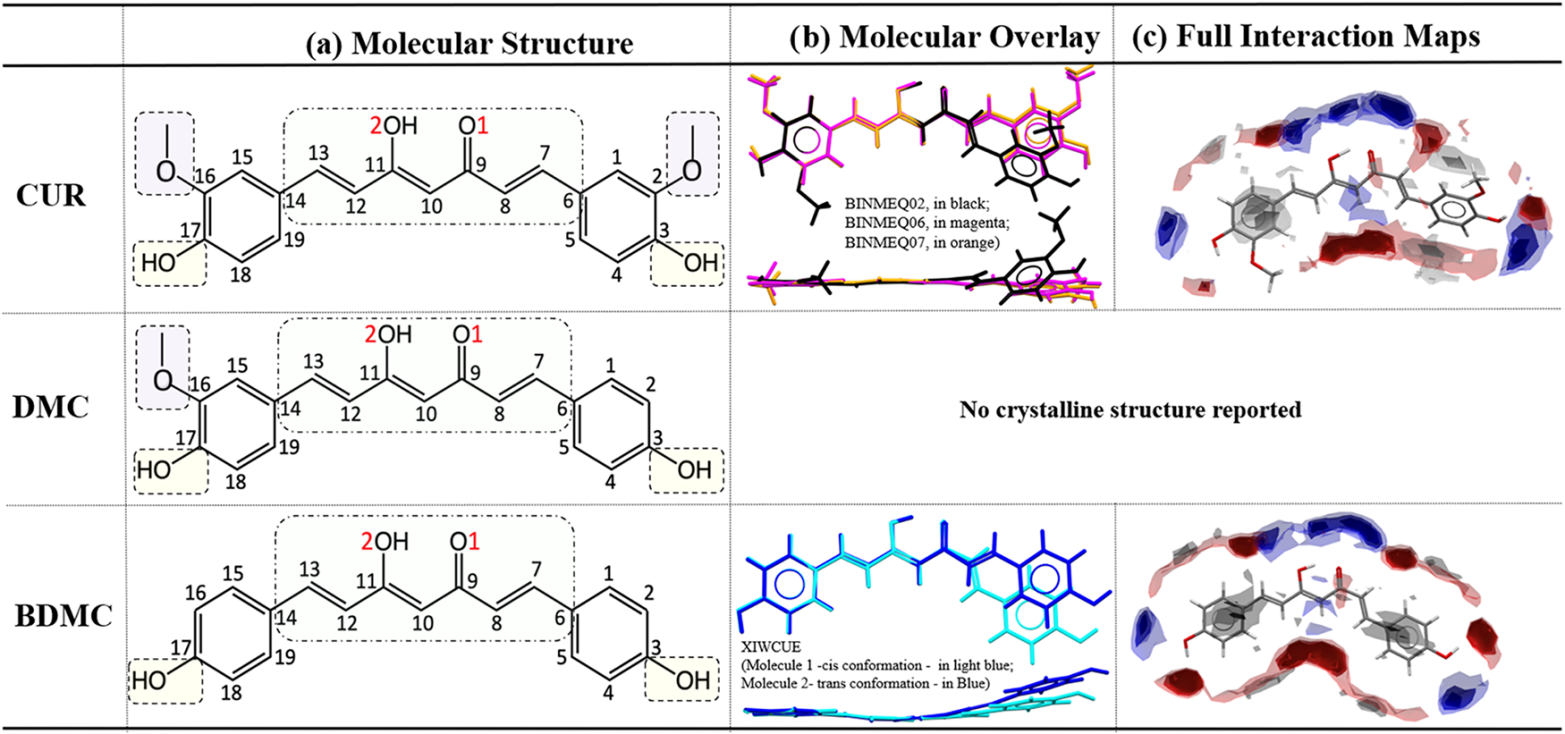
Comparison of (a) chemical diagram of CUR molecule with atom labels used in the conformational analysis of CUR, DMC and BDMC, (b) molecular conformations, showing two perpendicular projections with superpositions of the three CUR polymorphs and the two independent BDMC molecules, and (c) Full Interaction Maps (FIMs) highlighting the intermolecular interaction propensities of CUR (polymorph I) and BDMC (molecule in cis conformation) in their crystalline forms.

Regarding single component crystal structures, three polymorphs of CUR have been reported in the Cambridge Structural Database (CSD): polymorphs I, II, and III, with reference codes BINMEQ02, BINMEQ06, and BINMEQ07, respectively. Comparison of these structures reveals notable conformational differences, particularly between polymorph I and the other two forms. In polymorph I, the torsion angle C7–C8–C9–C10 adopts a cis conformation, whereas in polymorphs II and III it exhibits a trans conformation. Both methoxy groups in polymorphs II and III are oriented toward the keto–enol moiety, while in polymorph I the methoxy group in C16 is rotated away from it. Additionally, while in polymorphs II and III the molecules adopt nearly planar conformations, the phenyl ring bonded to carbon C7 is rotated by 44° in polymorph I (Figures 2b and 4).

When comparing the crystal structures of polymorphs II and III, both exhibit very similar packing arrangements, although with slight differences in molecular orientation. These differences can be visualized through the overlay of 15 molecules from each structure, where polymorph II is shown in gray (BINMEQ06) and polymorph III in green and red (BINMEQ07) depending the degree of deviation from the reference molecules of the former compound, as illustrated in Figure 3.

**Figure 3:**
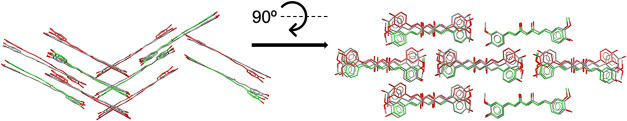
Superposition of 15 molecules from each crystal structure: polymorph II is shown in gray (BINMEQ06), and polymorph III in green and red (BINMEQ07), with color variation indicating the degree of deviation from the reference molecules of polymorph II.

For BDMC, only one crystalline form is currently reported in the Cambridge Structural Database (CSD), under the refcode XIWCUE. The structure contains two symmetry-independent BDMC molecules: one adopts a cis conformation at the C7–C8–C9–C10 torsion, while the other adopts a trans conformation. Neither molecule shows significant rotation of the phenyl rings; however, both exhibit slightly curved conformations along the main molecular axis (Figure 2b). Interestingly, in the crystal structure of BDMC, the molecules in cis conformation are arranged in well-defined layers, distinct from those formed by the trans conformers. The 2D molecular arrangement (substructure) within the cis layers is isostructural to the packing observed in polymorph I of CUR, Figure 4.

**Figure 4:**
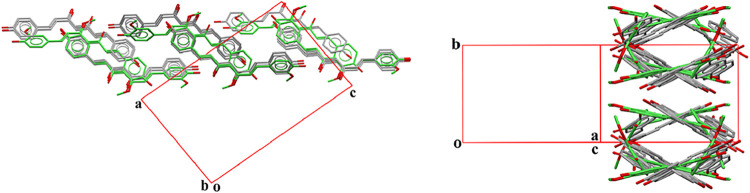
Superposition of molecular layers composed of cis-conformation BDMC molecules from the crystal structure of XIWCUE (shown in gray) with the equivalent molecular arrangement in curcumin polymorph I (BINMEQ02, shown in green) highlighting their isostructural 2D packing. The unit cell shown corresponds to the crystal structure of XIWCUE, and the views are projections along *b* axis and ac diagonal of this unit cell.

In the case of DMC, no single-component crystal structures have been reported to date.

FIM analyses of CUR and BDMC suggest that the keto–enolic and phenolic groups are the main hydrogen-bonding sites, mapped as red and blue regions that indicate high-probability positions for external donors and acceptors, respectively (Figure 2c). In contrast, the methoxy groups exhibit significant steric hindrance, and intramolecular hydrogen bonds between the methoxy oxygen and adjacent phenolic OH reduce the donor ability of these OH groups. The FIMs also display gray regions, highlighting favorable sites for hydrophobic contacts or π–π interactions on either side of the phenyl rings. Given the similarities observed between the FIMs of CUR and BDMC, the interaction landscape of DMC can be readily inferred, with the same hydrogen-bonding hotspots at the keto–enolic and phenolic groups and intermediate features arising from the presence of a single methoxy substituent.

These observations suggest that solvents containing hydrogen-bond donor or acceptor groups, such as ketones, ethers, and amines, are likely to interact with the hydrogen-bonding sites of curcuminoids (hydroxy and keto–enolic groups), potentially promoting solvate formation.

#### 3.1.1 Reported Solvates of Curcumin and Its Derivatives

Crystal structures of CUR solvates with 1,4-dioxane (DOX) (LADXEX, LADXIB),^15^ acetone (ACE) (FIHRUN),^14^ and dichloromethane (DCM) (OJIWOV),^13^ as well as BDMC solvates with isopropanol (ISP) (XIWDEP),^16^ acetone (ACE) (XIWDAL),^16^ methanol (MET) (BUWKUZ),^16^ and water (GANJAG),^12^ have been reported in the Cambridge Structural Database (CSD). In addition, the literature describes evidence of CUR solvates with methyl acetate (MAC)^11^ and water,^10^ and BDMC solvates with DOX,^17^ tetrahydrofuran (THF),^17^ and dimethyl sulfoxide (DMSO);^17^ however, their crystal structures have not yet been determined. No solvate forms have been reported for DMC to date.

All curcuminoid molecules adopt a trans conformation at the C7–C8–C9–C10 dihedral angle in their crystal structures, with the exception of the CUR–DCM solvate, which contains two symmetry-independent CUR molecules, both exhibiting a cis conformation (Figures 5 and 6).

**Figure 5:**
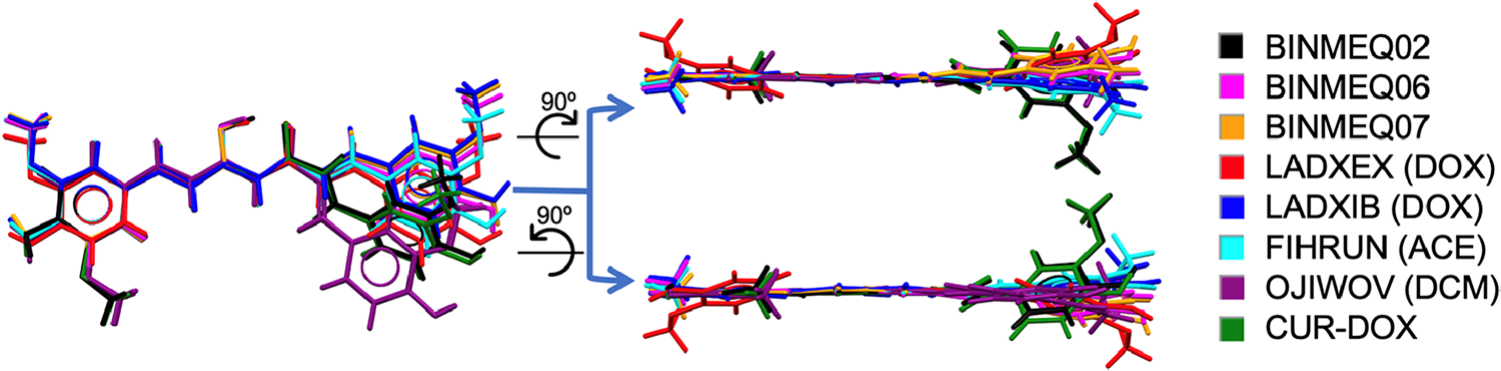
Superposition of curcumin (CUR) molecules from all known crystal structures, including polymorphs I, II, and III (BINMEQ02,06,07), previously reported solvates from the Cambridge Structural Database (CSD), and the new solvate structure obtained in this work (CUR-DOX). Each structure is shown in a different color, as indicated in the accompanying legend.

**Figure 6:**
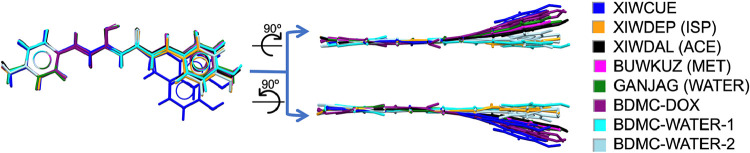
Superposition of bisdemethoxycurcumin (BDMC) molecules from all available crystal structures, including reported solvates in the Cambridge Structural Database (CSD) and the new solvates identified in this work (BDMC-DOX, BDMC-WATER-1, BDMC-WATER-2). Each structure is represented in a different color, as indicated in the accompanying legend.

#### 3.1.2 New Solvates Forms of CUR, DMC and BDMC and Comparative Crystal Structure Analysis with Previously Reported Structures

As described in the Methods, 41 solvents were screened for CUR, and 16 for DMC and BDMC due to limited material availability (Table S3). Single crystals of CUR, DMC, and BDMC were successfully obtained using 1,4-dioxane (DOX) as crystallization solvent.

For BDMC, two distinct hydrated forms were isolated from the same recrystallization in tetrahydrofuran (THF), here designated BDMC-WATER-1 and BDMC-WATER-2. A THF solvate of BDMC had previously been reported without structural characterization.^17^ Comparison of the PXRD pattern provided by those authors with the present phases confirms that BDMC-WATER-1 and BDMC-WATER-2 are different crystalline forms. The earlier report therefore most likely corresponds to a distinct hydrate or a genuine THF solvate (Figure S1). The two hydrates crystallized simultaneously in the same vessel as morphologically distinct ribbon-like crystals, one yellow and one orange, each associated with a different structure (Figure S2).

Notably, this work reports the first DMC solvate structure, obtained with DOX (DMC-DOX), representing the first crystal structure of DMC described in the literature. Crystals of CUR (from DMSO) and DMC (from ACN) were also obtained, although only unit cell parameters could be determined as the crystals degraded before full diffraction data sets could be collected.

The molecular structures of the new crystalline forms are presented in Figure 7, with displacement ellipsoids drawn at the 50% probability level for non-hydrogen atoms. Complete crystallographic and refinement data are provided in Table S4 and have been deposited with the Cambridge Crystallographic Data Centre (CCDC).

**Figure 7:**
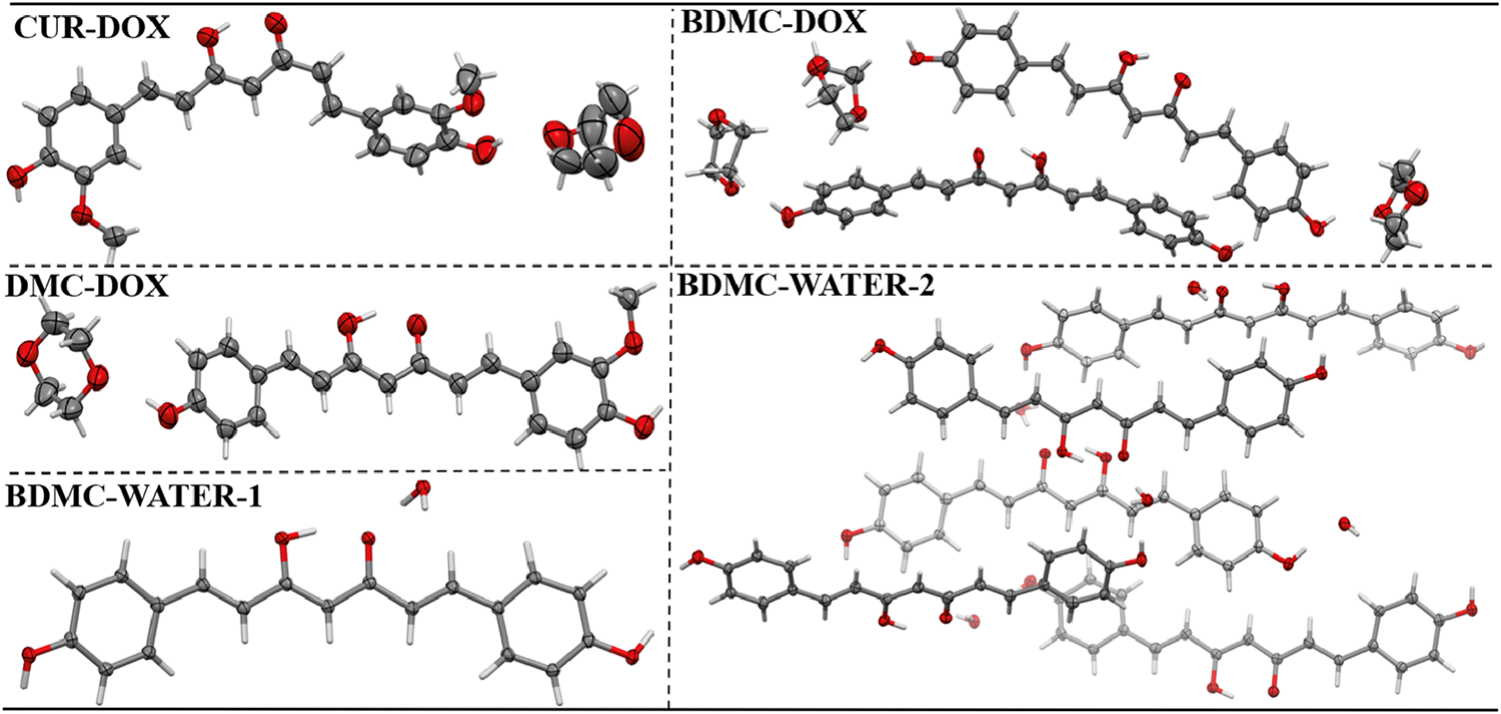
Molecular structures of CUR-DOX, DMC-DOX, BDMC-DOX, BDMC-WATER-1, BDMC-WATER-2 showing thermal ellipsoids with 50% probability (except the hydrogen atoms). The disorder of DOX molecule in CUR-DOX and BDMC-DOX was omitted just for clarity purposes.

#### 3.1.3 CUR-DOX

Besides the two previously reported 1,4-dioxane solvates of curcumin (CUR-DOX: LADXEX and LADXIB) in the CSD, we have crystallized a new 1,4-dioxane solvate in the present work. In both reported CUR-DOX solvates, the CUR molecules adopt a trans conformation in their conjugated chain linking the aromatic rings. In contrast, the CUR molecules in the new solvate exhibit a cis conformation, similar to that observed in polymorph I of CUR. Additionally, the phenyl ring attached to carbon C7 is rotated by 52° relative to the plane of the rest of the molecule, and the conformation matches that of CUR in polymorph I, as shown by the perfect overlap (Figure 5, green and black molecules, respectively).

This structure exhibits a 1:1 molecular ratio of CUR to 1,4-dioxane, with the solvent modeled as disordered. LADXEX also contains one independent molecule in a 1:1 ratio, while LADXIB shows a 1:1.5 ratio. No similarity was found in the molecular packing of the three DOX solvates, except for the common tape motifs observed in both LADXEX and LADXIB (Figure 8a) but not in CUR-DOX, where the molecules grow through stacking of the planar part of the structure, extending into 2D layers stabilized by O6–H···O1=C and C8–H···O1=C hydrogen bonds (D···A distances of 2.791(3) Å and 3.736(4) Å respectively), Figure 8b.

**Figure 8:**
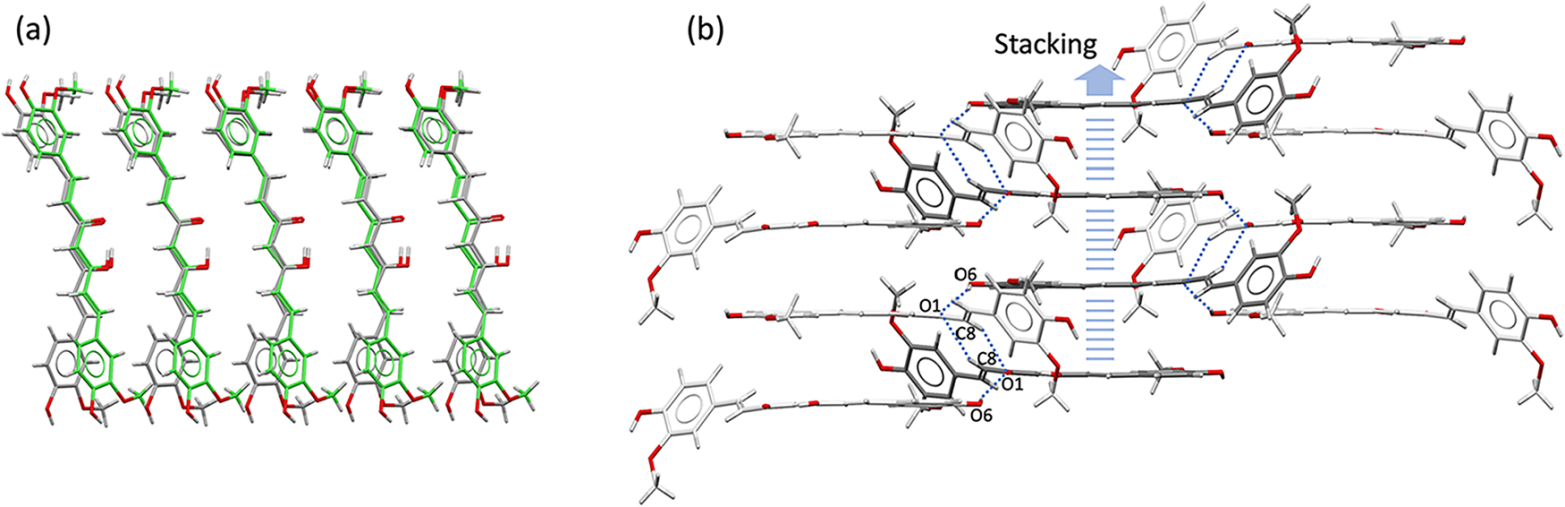
(a) Superposition of molecules from the crystal structures of LADXEX (gray) and LADXIB (green), highlighting the common tape motifs observed in both structures. (b) Tapes in CUR-DOX formed through the stacking of the planar delocalized bonds in the CUR molecule, and 2D structures stabilized by hydrogen bonds.

#### 3.1.4 DMC-DOX

This is the first crystal structure determination of the DMC molecule. In its solvate form (DMC-DOX), DMC adopts a trans conformation, with the methoxy group retained on the keto side of the molecule and oriented toward the keto–enol moiety. The molecule exhibits a highly curved geometry along its main molecular axis.

The crystal structure of DMC reveals tape-like arrangements similar to those observed in LADXEX and LADXIB (Figure 8). Moreover, these tapes extend into two-dimensional architectures equivalent to those seen in LADXIB (Figure 9) through weak hydrogen bonds CH···pi at 3.528(9) Å.

**Figure 9:**
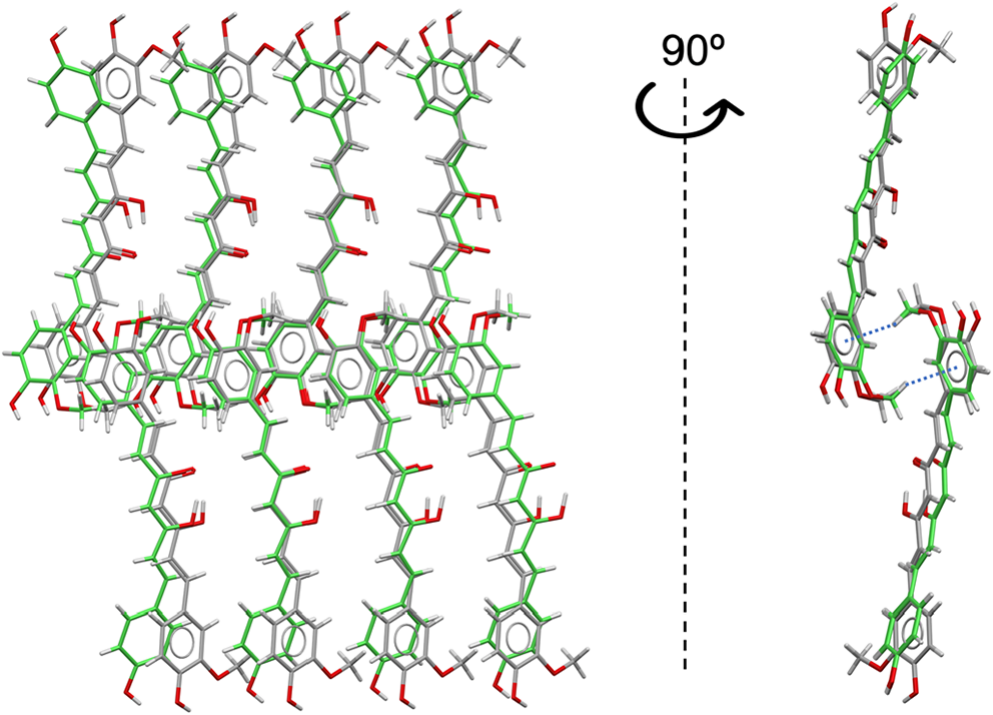
Superposition of molecules from the crystal structures of LADXIB (in gray) and DMC-DOX (in green), highlighting the common tape motifs and equivalent two-dimensional arrangements observed.

#### 3.1.5 BDMC-DOX

In the crystal structure of the BDMC–DOX solvate, the asymmetric unit contains two BDMC molecules and three 1,4-dioxane (DOX) molecules. Each BDMC molecule adopts a planar trans conformation and independently forms molecular tapes (Figure 10a), analogous to those observed in previously reported DOX solvates of CUR derivatives (LADXEX, LADXIB, and DMC–DOX).

**Figure 10:**
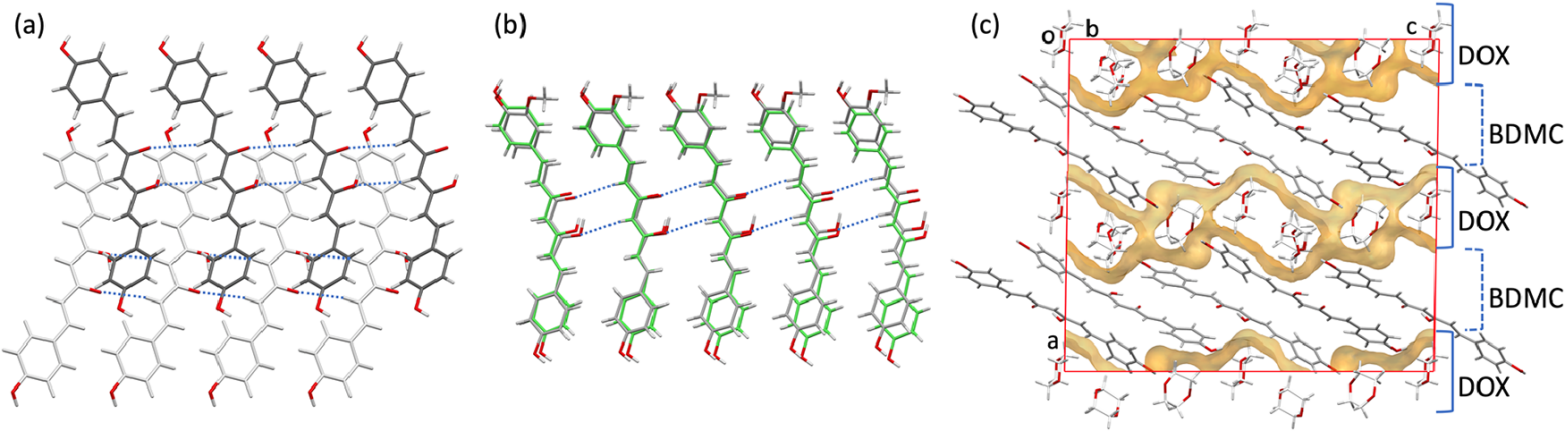
(a) Molecular tapes formed by the two independent molecules in BDMC-DOX structure (one shown in dark gray and the other in light gray). (b) Superposition of molecules from the crystal structures of DMC-DOX (gray) and BDMC-DOX (green), highlighting their common tape motifs. (c) Projections along the ***b*** axis of BDMC-DOX showing the layered packing with alternating two-dimensional sheets of BDMC and DOX molecules.

In all four DOX solvates, the molecular tapes result from translational repetition along the *b*-axis. The lengths of the *b*-axis for each structure are as follows: LADXEX, 5.346 Å; LADXIB, 5.418 Å; DMC–DOX, 5.6502(4) Å; and BDMC–DOX, 5.7592(7) Å. These tapes are stabilized by weak C–H···O hydrogen bonds between the methylene protons of the central aliphatic linker and the oxygen atoms of the keto–enol moieties of adjacent molecules.

The instability of BDMC–DOX crystals, already noted above, can be understood in light of their layered architecture. The structure consists of alternating two-dimensional sheets of BDMC and DOX molecules, with one of the three independent DOX molecules forming strong hydrogen bonds that bridge adjacent BDMC layers, Figure 10c and Table S3. Nevertheless, the extended segregation of solvent into continuous layers reduces the overall robustness of the packing, making the crystals particularly sensitive to ambient conditions.

#### 3.1.6 BDMC-WATER

Two hydrated forms of BDMC were crystallized in this work, both with a 1:1 stoichiometric ratio of BDMC to water. BDMC-WATER-1 contains one molecule in the asymmetric unit (*Z*′ = 1), whereas BDMC-WATER-2 contains five independent molecules (*Z*′ = 5). In both cases, the BDMC molecules adopt a trans conformation along the central linker, with no significant differences in molecular geometry. A previously reported hydrate of BDMC (CSD refcode GANJAG) also shows a 1:1 stoichiometry and *Z*′ = 1. This structure and BDMC-WATER-1 are isostructural at the level of molecular layers (Figure 11), although the stacking between layers differs significantly.

**Figure 11:**
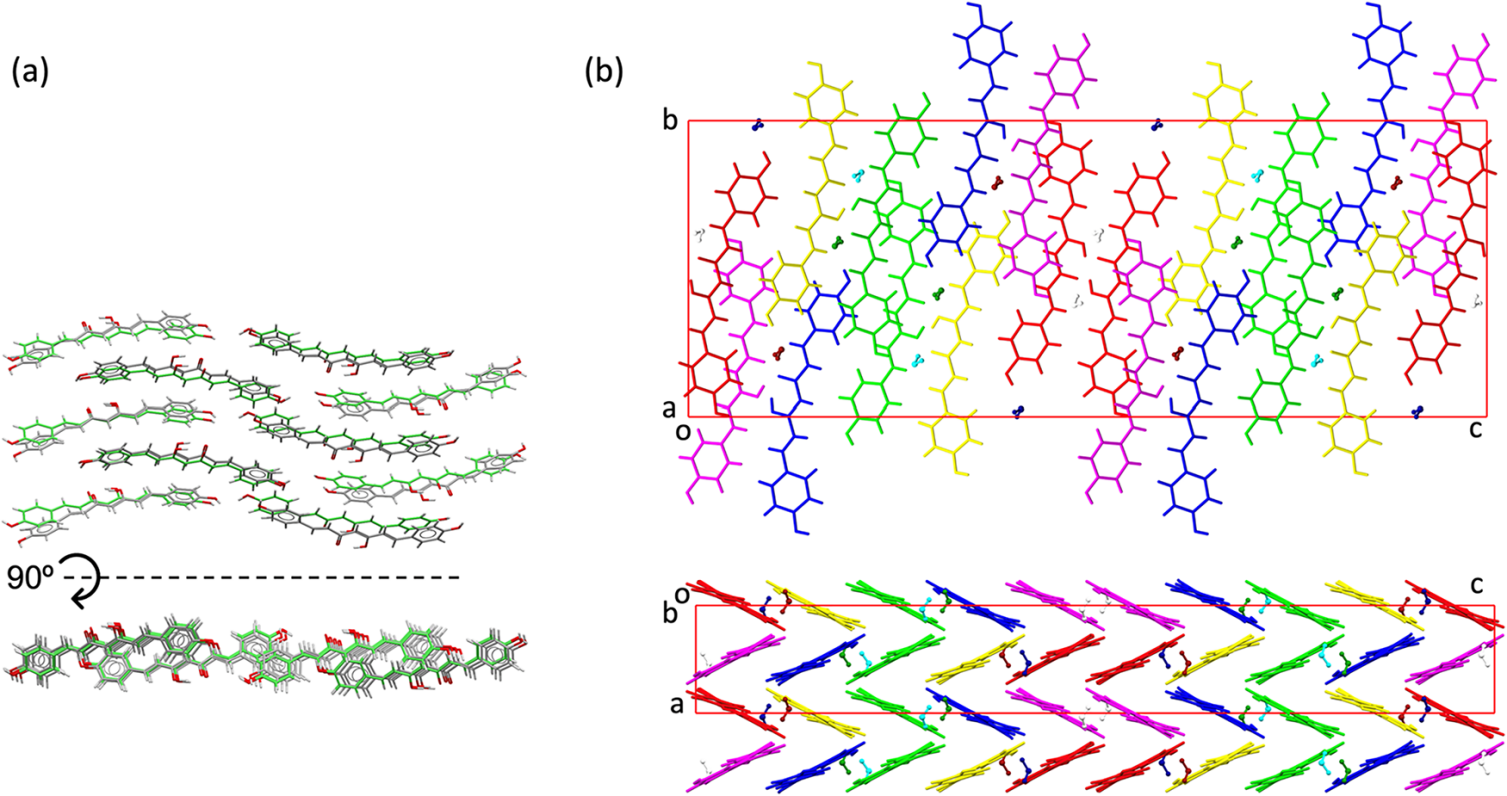
(a) Superposition of molecules from the crystal structures of GANJAG (gray, hydrate) and BDMC-Water-1 (green), showing their common 2D structural motifs. (b) Projections along the ***a*** (top) and ***b*** (bottom) axes of the crystal structure of BDMC-Water-2, with each independent molecule shown in a different color.

Interestingly, a comparable layered arrangement is also found in the methanol solvate of BDMC (BUWKUZ). Moreover, BDMC–methanol (BUWKUZ) and BDMC–water (GANJAG) are 3D isostructural (Figure 12), displaying nearly identical superpositions of layers. The key difference is that in BUWKUZ the layers are distorted relative to each other, owing to the larger size of methanol molecules, which increases the separation between BDMC layers. Hydrogen-bonding patterns reflect this substitution effect. In GANJAG, each water molecule participates in four hydrogen bonds, acting twice as donor and twice as acceptor. When water is replaced by methanol in BUWKUZ, three of these interactions are retained: the hydroxyl group of methanol accepts two hydrogen bonds and donates one (Table S3). The second donor interaction, however, is lost, precisely along the direction in which the structure becomes elongated.

**Figure 12:**
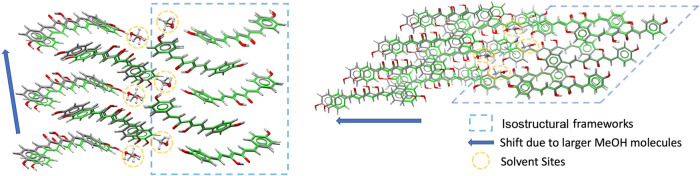
Superposition of molecules from the crystal structures of BUWKUZ (gray, methanol solvate) and GANJAG (green, hydrate), showing their 3D isostructurality. The circled regions highlight the solvent sites, where accommodation of the bulkier MeOH molecules induces a relative displacement of adjacent layers.

The crystal structure of BDMC-Water-2 differs from any of the structures solved to date, Figure 11b.

### 3.2 Analyses of Statistical and Thermodynamic Prediction Methods Using for Obtaining Solvates

The prediction results for obtaining CUR, DMC and BDMC solvates using statistical and thermodynamic methods are presented in summary Table 1 and in more detail in Tables S5–S7 of the Supporting Information. In the crystallization results, new solvates and hydrates were obtained for CUR, BDMC and DMC. These new solvates, CUR-DOX, BDMC-DOX, DMC-DOX, BDMC-WATER1, BDMC-WATER2 (with determined crystal structure) and CUR-DMSO and DMC-ACN (with unit cell parameter information) were included in the evaluation of the prediction methods, as well as the solvates already described in the literature.

**Table 1:**
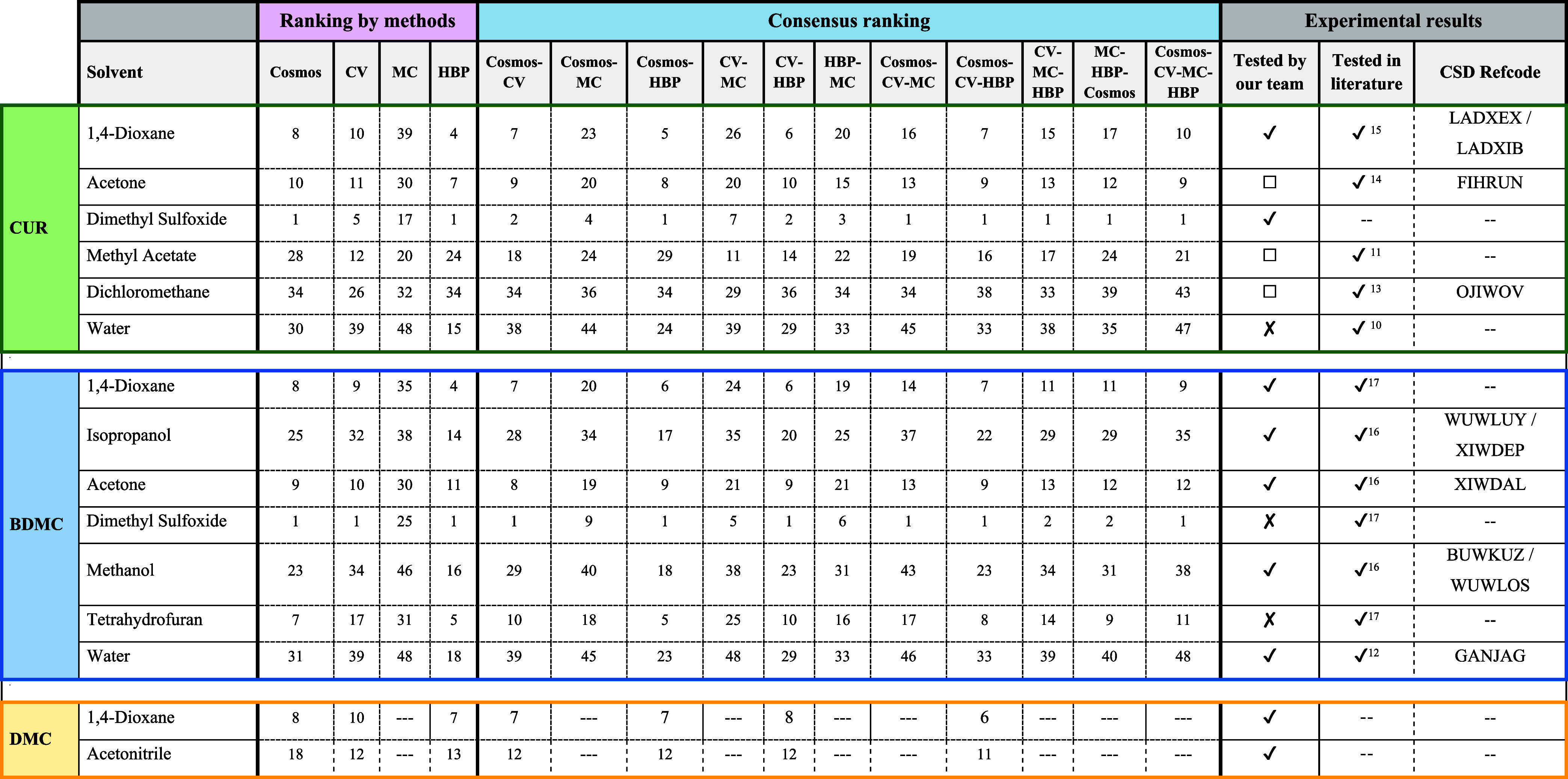
Ranking and Consensus Ranking Positions of **New Solvates** Prepared in This Work and in the **Literature Reported** for Curcumin (CUR), Bisdemethoxycurcumin (BDMC), and Demethoxycurcumin (DMC), Obtained through Statistical (MC, CV, HBP) and Thermodynamic (COSMO) Methods^a^

a The √ indicates that the solvent was tested and the solvate form was obtained. The *X* indicates that the solvent was tested, but the solvate form was not obtained (just precipitate). The □ indicates that the solvent was tested, resulting in the formation of polymorph I.

Since CUR and BDMC exhibit a larger number of solvates, the analysis of the prediction methods was first carried out for these molecules, followed by the assessment of DMC. Based on the evaluation of each individual and combined method, the **HBP** approach gave the best results for solvate prediction, as summarized in Table S8. This outcome indicates that hydrogen bonding is the predominant interaction driving solvate formation in CUR and BDMC. This is further corroborated by Table 2, which shows that nearly all solvent molecules form strong hydrogen bonds with the hydroxy and keto–enolic groups of CUR, DMC, or BDMC. The only exception is the CUR–dichloromethane solvate, which does not exhibit strong hydrogen bonds but instead multiple weak interactions of the C–H···O and C–H···Cl types. These weaker contacts are not well captured by the HBP model, which explains the anomalously low ranking obtained for this solvate.

**Table 2:** Hydrogen Bonds between Solvent Molecules and the Host Structures, with Distances Given between the Non-Hydrogen Atoms Involved (a and b Denote Molecules in a Disorder Model; Subscripts A, B, C, D and E Indicate Independent Molecules in the Structures)

compounds	solvent-(CUR, DMC, BDMC) interactions	type of interaction	distances (Å)	compounds	solvent-(CUR, DMC, BDMC) interactions	type of interaction	distances (Å)
CUR							
LADXEX (DOX)	Yes	O4–H···O_DOX_	2.676(4)	CUR-DOX	Yes	O3–H···O_DOX_	a: 2.83(1), b: 2.72(2)
		O6–H···O_DOX_	2.665(4)			C20–H_3_···O_DOX‑a_	3.44(1)
						C20–H_3_···O_DOX‑b_	3.62
LADXIB (DOX)	Yes	O4–H···O_DOX‑A_	2.666(2)	OJIWOV (CH_2_Cl_2_)	It does not present strong HBs but a lot of weak HBs, CH···O and CH···Cl type. These HBs are not well represented in HBP what is reflected in the high values that this molecule obtains in his ranking		
		O6–H···O_DOX‑B_	2.691(2)				
BDMC							
BDMC-DOX	Yes	O4–H_A_···O_DOX‑A_	2.62(3)	BDMC-Water-2	Yes	O_WA_-H···O1_A_	2.780(2)
		O4–H_B_···O_DOX‑A_	2.77(3)			O_WA_-H···O2H_C_	2.710(2)
		O3–H_B_···O_DOX‑B_	2.70(2)			O3_E_-H··· O_WA_	2.774(2)
		O3–H_A_···O_DOX‑C_	a: 2.72(4)			O4_E_-H··· O_WA_	2.713(2)
			b: 2.73(3)			O_WB_-H···O1_B_	2.701(2)
XIWDEP (^i^PrOH)	Yes	O3–H···O_iso‑PROP_	2.640(3)			O_WB_-H···O2H_D_	2.778(2)
		O_iso‑PROP_-H···O3H	2.759(3)			O3_A_-H··· O_WB_	2.772(2)
XIWDAL (Acetone)	Yes	O3–H···O_Ace_	2.761(4)			O4_A_-H··· O_WB_	2.727(2)
		C_Ace_-H_3_···O2H	3.536(7)			O_WC_-H···O1_D_	2.702(2)
		C_Ace_-H_3_···O4H	3.328(6)			O_WC_-H···O2H_B_	2.781(2)
BUWKUZ (MeOH)	Yes	O_MeOH_-H···O1	2.716			O3_B_-H··· O_WC_	2.780(2)
		O3–H··· O_MeOH_	2.758			O4_B_-H··· O_WC_	2.716(2)
		O4–H··· O_MeOH_	2.719			O_WD_-H···O1_C_	2.700(2)
BDMC-Water-1	Yes	O_w_-H···O1	2.751(1)			O_WD_-H···O2H_A_	2.774(2)
		O_w_-H···O1	2.801(2)			O3_D_-H···O_WD_	2.785(2)
		O4–H···O_w_	2.754(2)			O4_D_-H···O_WD_	2.714(2)
		O3–H···O_w_	2.796(1)			O_WE_-H···O1_E_	2.700(2)
GANJAG (Water)	Yes	O_w_-H···O1	2.735(3)			O_WE_-H···O2H_E_	2.755(2)
		O_w_-H···O3	2.756(4)			O3_C_-H···O_WE_	2.779(2)
		O3–H···O_w_	2.742(4)			O4_C_-H···O_WE_	2.720(2)
		O4–H···O_w_	2.742(4)				
DMC							
DMC-DOX	Yes	O4–H···O_DOX_	2.724(8)				
		O5–H···O_DOX_	2.689(8)				

When analyzing the CV method, which evaluates the acceptor and donor capacity of the atoms of the functional group of each molecule, its performance was affected by the accessibility of these functional groups. Although several conformations of both the target molecule and the solvent (when was applicable) were generated in an attempt to improve accessibility between the molecules, the prediction values did not improve. Regarding the COSMO method, it was observed that its combination with HBP provided better prediction results compared to the individual method.

The worst performing solvate prediction method was MC, both as a single technique and in combination with other techniques. It was observed that the positions of CUR and BDMC solvates appeared at the bottom of the ranking, suggesting that solvates would not form. The differences in shape, size and molecular polarity of the molecules and accessibility between the molecules could have had a significant impact on the evaluation of solvate formation. To improve accessibility between molecules, different conformations were generated for all molecules; however, the prediction results showed no improvement.

For DMC, no solvates had been previously reported. In this work, two new solvates were obtained: one with a fully solved crystal structure and another characterized only by unit cell parameters. As observed for CUR and BDMC, the HBP method provided the most accurate ranking for these new solvates when compared with the other prediction methods. Since the MC approach proved unsuitable for solvate prediction in CUR and BDMC, it was not applied to DMC.

## 4 Conclusion

This study provides a systematic comparison between statistical and thermodynamic methods for predicting solvate formation in CUR and its derivatives DMC and BDMC. Through an extensive crystallization screen, several new solvates and hydrates were identified, including the first crystal structure of DMC, as well as novel BDMC hydrates and additional CUR and BDMC solvates.

Evaluation of predictive methodologies demonstrated that the Hydrogen Bond Propensity (HBP) method outperformed other statistical approaches, consistently ranking experimental solvates near the top of its predictions. The COSMO-RS thermodynamic model, while less accurate on its own, showed enhanced performance when combined with HBP, highlighting the benefit of hybrid prediction strategies. In contrast, the Molecular Complementarity (MC) method proved unsuitable for solvate screening, as it often misranked known solvates.

The findings also emphasize the critical role of hydrogen bonding as the primary driving force in curcuminoid solvate formation. Nearly all experimentally obtained solvates involved strong hydrogen-bond interactions with solvent molecules, with the exception of CUR–dichloromethane, which was stabilized instead by multiple weaker contacts. Such cases underscore the need for predictive models to better capture the contribution of weaker interactions.

Overall, this work not only expands the structural knowledge of curcuminoid solvates but also benchmarks the relative strengths and weaknesses of current predictive methods. The combined HBP–COSMO approach emerges as the most reliable strategy, offering valuable guidance for rational solvate screening in pharmaceuticals and functional materials.

## Supplementary Material



## Data Availability

Data will be made available on request.

## Notes

PXRD patterns of the BDMC-THF solvate reported in the literature and the two title hydrates; information on the solvents used in the prediction methods and experimental crystallisation screening of CUR, BDMC, and DMC; crystallographic information for crystal data and structure refinement of new solvates and hydrates for CUR, BDMC, and DMC; list of all solvents tested with CUR, BDMC, and DMC to form solvates (PDF)
