# Supplementary material for: Comparative Assessment of Statistical and Thermodynamic Prediction Methods for Solvate Formation: A Case Study with Curcumin and Its Derivatives
Source: Cryst Growth Des. 2025 Dec 9;26(1):390–400. doi: 10.1021/acs.cgd.5c01343 (PMC12787670; doi:10.1021/acs.cgd.5c01343)
Supplement: Supplementary file 1 [file cg5c01343_si_001.pdf]

Supplementary Information (SI)

Comparative Assessment of Statistical and Thermodynamic Prediction Methods for Solvate Formation: A Case Study with Curcumin and Its Derivatives

Julian Ticona-Chambi[1], Duane Choquesillo-Lazarte[2], Silvia Lucia Cuffini[1], Lourdes Infantes[3]

[1]Instituto de Ciência e Tecnologia (ICT), Universidade Federal de São Paulo (UNIFESP), Brazil.

[2]Laboratorio de Estudios Cristalográficos, IACT-CSIC, Avda. de las Palmeras 4, 18100 Armilla, Spain

[3] Instituto de Química y Física Bass Cabrera (IQF), Consejo Superior de Investigaciones Científicas (CSIC), Madrid, Spain.

*Corresponding author e-mail: xlourdes@iqf.csic.es

Tables and Figures of Contents:

Figure S1 PXRD patterns of the BDMC-THF solvate reported in the literature and the two hydrates (BDMC-WATER-1 and BDMC-WATER-2) obtained in this work.

Figure S2 Single crystals of new solvates and hydrates for CUR, DMC, and BDMC.

Table S1. Information on the solvents used in the prediction methods and experimental crystallisation screening of CUR, BDMC and DMC. CID, CAS, Molecular Formula, Molecular Weight and SMILE were obtained from PubChem database (https://pubchem.ncbi.nlm.nih.gov/).

Table S2. Crystallization experiments to identify new solvates and hydrates of CUR, DMC and BDMC.

Table S3. New solvate and hydrate forms for CUR, DCM and BDCM identified by co-crystallization experiments.

Table S4. Crystallographic information for crystal data and structure refinement of new solvates and hydrates for CUR, BDMC, and DMC.

Table S5 List of all solvents tested with curcumin (**CUR)** to form solvates. Ranking and Consensus Ranking positions obtained through statistical (MC, CV, HBP) and thermodynamic (COSMO) methods.. The ✗ indicates that the solvent was tested, but the solvate form was not obtained (just precipitate). The ☐ indicates that the solvent was tested, resulting in the formation of polymorph I. The --- indicates that the solvent was not tested. The ✓ indicates that the solvate was tested and the solvate form was obtained. New solvates obtained in this work and those reported in the literature are highlighted in bold green.

Table S6 List of all solvents tested with bisdemethoxycurcumin (**BDMC)** to form solvates. Ranking and Consensus Ranking positions obtained through statistical (MC, CV, HBP) and thermodynamic (COSMO) methods.. The ✗ indicates that the solvent was tested, but the solvate form was not obtained (just precipitate). The ☐ indicates that the solvent was tested, resulting in the formation of polymorph I. The **---** indicates that the solvent was not tested. The ✓ indicates that the solvate was tested and the solvate form was obtained. New solvates obtained in this work and those reported in the literature are highlighted in bold green.

Table S7 List of all solvents tested with demethoxycurcumin (**DMC)** to form solvates. Ranking and Consensus Ranking positions obtained through statistical (MC, CV, HBP) and thermodynamic (COSMO) methods.. The ✗ indicates that the solvent was tested, but the solvate form was not obtained (just precipitate). The ☐ indicates that the solvent was tested, resulting in the formation of polymorph I. The **---** indicates that the solvent was not tested. The ✓ indicates that the solvate was tested and the solvate form was obtained. New solvates obtained in this work and those reported in the literature are highlighted in bold green.

Table S8 Comparison of the ranking and consensus ranking of prediction methods for solvates.

Worked-out example

A worked example for the calculation of ΔCV for each component: the target molecule (IFA), the solvent, and the multicomponent system (IFA–solvent).

Supplementary Information (SI)

Figure S1 PXRD patterns of the BDMC-THF solvate reported in the literature and the two hydrates (BDMC-WATER-1 and BDMC-WATER-2) obtained in this work

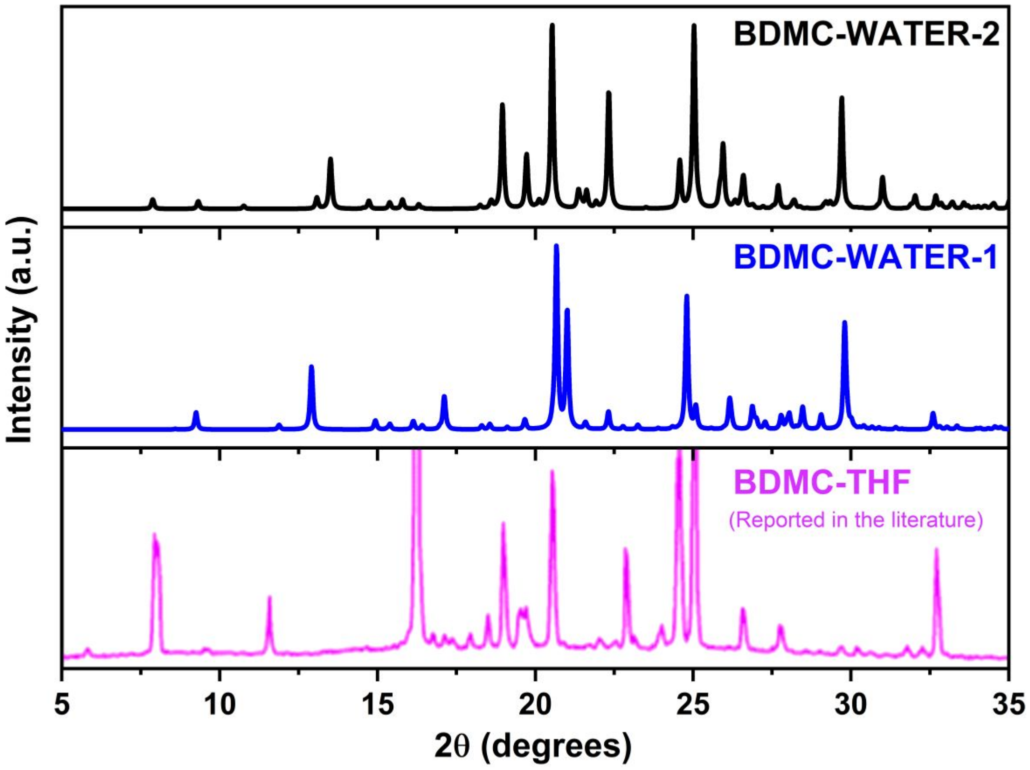

Figure S2 Single crystals of new solvates and hydrates for CUR, DMC, and BDMC.

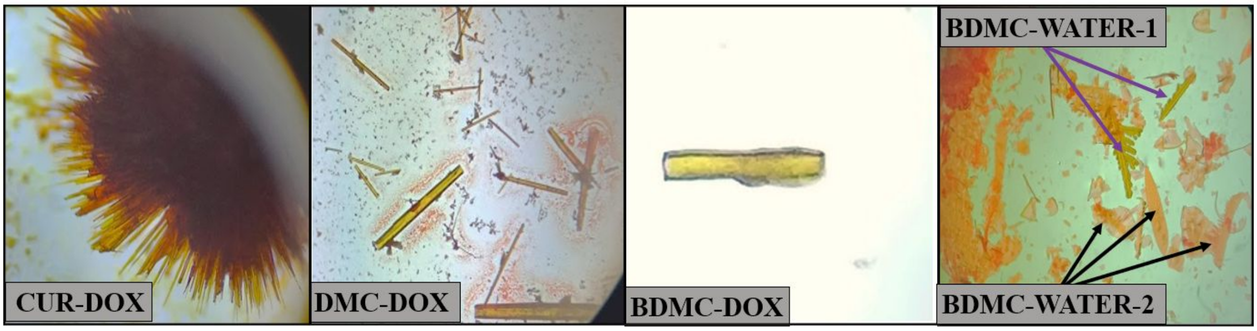

Table S1. Information on the solvents used in the prediction methods and experimental crystallisation screening of CUR, BDMC and DMC. CID, CAS, Molecular Formula, Molecular Weight and SMILE were obtained from PubChem database (https://pubchem.ncbi.nlm.nih.gov/).

Substance	CID	Acronym	CAS	Formula	Molecular Weight (g/mol)	SMILE	Molecule used in predictive analysis	Molecule used in experimental crystallization
Curcumin (Tautomer 1)	969516	CUR	458-37-7	$C_{21}H_{20}O_6$	368.4	OC1=CC=C(/C=C/C(/C=C(O)/C=C/C2=CC(OC)=C(O)C=C2)=O)C=C1OC	✓	✓
Curcumin (Tautomer 2)						OC1=CC=C(/C=C/C(CC(/C=C/C2=CC(OC)=C(O)C=C2)=O)=O)C=C1OC		
Bisdemethoxycurcumin (Tautomer 1)	5315472	BDMC	33171-05-0	$C_{19}H_{16}O_4$	308.3	OC1=CC=C(/C=C/C(/C=C(O)/C=C/C2=CC=C(O)C=C2)=O)C=C1	✓	✓
Bisdemethoxycurcumin (Tautomer 2)						OC1=CC=C(/C=C/C(CC(/C=C/C2=CC=C(O)C=C2)=O)=O)C=C1		
Demethoxycurcumin (Tautomer 1)	5469424	DMC	22608-11-3	$C_{20}H_{18}O_5$	338.4	OC1=CC=C(/C=C/C(/C=C(O)/C=C/C2=CC=C(O)C(OC)=C2)=O)C=C1	✓	✓
Demethoxycurcumin (Tautomer 2)						OC1=CC=C(/C=C/C(CC(/C=C/C2=CC(OC)=C(O)C=C2)=O)=O)C=C1		
1,4-Dioxane	31275	DOX	123-91-1	$C_4H_8O_2$	88.1	C1COCCO1	✓	✓
1-Butanol	263	BUT	71-36-3	$C_4H_{10}O$	74.1	CCCCO	✓	✓
1-Hexanol	8103	6OH	111-27-3	$C_6H_{14}O$	102.2	CCCCCCO	✓	✓
1-Octanol	957	8OH	111-87-5	$C_8H_{18}O$	130.2	CCCCCCCCO	✓	✓
1-Pentanol	6276	5OH	71-41-0	$C_5H_{12}O$	88.1	CCCCCO	✓	✓
1-Propanol	1031	PROH	71-23-8	C_3H_8O	60.1	CCCO	✓	✓
2-Methylfuran	10797	METFRU	534-22-5	C_5H_6O	82.1	CC1=CC=CO1	✓	---
2-Propanol	3776	ISP	67-63-0	C_3H_8O	60.1	CC(C)O	✓	✓
Acetic Acid	176	ACT	64-19-7	$C_2H_4O_2$	60.1	CC(=O)O	✓	✓
Acetone	180	ACE	67-64-1	C_3H_6O	58.1	CC(=O)C	✓	✓
Acetonitrile	6342	ACN	75-05-8	C_2H_3N	41.1	CC#N	✓	✓
Acetophenone	7410	ACP	98-86-2	C_8H_8O	120.1	CC(=O)C1=CC=CC=C1	✓	✓
Benzene	241	BZN	71-43-2	C_6H_6	78.1	C1=CC=CC=C1	✓	✓
Chlorobenzene	7964	CLB	108-90-7	C_6H_5Cl	112.6	C1=CC=C(C=C1)Cl	✓	✓
Chloroform	6212	CLF	67-66-3	$CHCl_3$	119.4	C(Cl)(Cl)Cl	✓	✓
Cyclohexane	8078	CLH	110-82-7	C6H12	84.2	C1CCCCC1	✓	✓
Cyclopentane	9253	CLP	287-92-3	C_5H_{10}	70.1	C1CCCC1	✓	✓
Cyclohexanone	7967	CHEXONE	108-94-1	$C_6H_{10}O$	98.14	C1CCC(=O)CC1	✓	---
Cyclopentanone	8452	CICLOPEN	120-92-3	C_5H8O	84.1	C1CCC(=O)C1	✓	✓
Diethyl Ether	3283	DET	60-29-7	$C_4H_{10}O$	74.1	CCOCC	✓	✓

Table S1 (continued)

Substance	CID	Acronym	CAS	Formula	Molecular Weight (g/mol)	SMILE	Molecule used in predictive analysis	Molecule used in experimental crystallization
Dimethyl Formamide	6228	DMF	68-12-2	C_3H_7NO	73.1	CN(C)C=O	✓	✓
Dimethyl Sulfide	1068	DMSE	75-18-3	C_2H_6S	62.1	CSC	✓	---
Dimethyl Sulfoxide	679	DMSO	67-68-5	C_2H_6OS	78.1	CS(=O)C	✓	✓
Ethanol	702	EtOH	64-17-5	C_2H_6O	46.1	CCO	✓	✓
Ethyl Acetate	8857	ETA	141-78-6	$C_4H_8O_2$	88.1	CCOC(=O)C	✓	✓
Ethylene	6325	ETHEN	74-85-1	C_2H_4	28.1	C=C	✓	---
Formic Acid	284	FACID	64-18-6	CH_2O_2	46.0	C(=O)O	✓	---
Heptane	8900	HEP	142-82-5	C_7H_{16}	100.2	CCCCCCC	✓	✓
Hexane	8058	HEX	110-54-3	C_6H_{14}	86.2	CCCCCC	✓	✓
Isopropyl Ether	7914	DIE	108-20-3	$C_6H_{14}O$	102.2	CC(C)OC(C)C	✓	✓
m-Cresol	342	MCRES	108-39-4	C_7H_8O	108.1	CC1=CC(=CC=C1)O	✓	✓
Methanol	887	MET	67-56-1	CH_4O	32.0	CO	✓	✓
Methyl Acetate	6584	MAC	79-20-9	$C_3H_6O_2$	74.1	CC(=O)OC	✓	✓
Methyl Ethyl Ketone	6569	MEK	78-93-3	C_4H_8O	72.1	CCC(=O)C	✓	✓
Methylene Dichloride	6344	DCM	75-09-2	CH_2Cl_2	84.9	C(Cl)Cl	✓	✓
N,N-Dimethyl Acetamide	31374	DMA	127-19-5	C_4H_9NO	87.1	CC(=O)N(C)C	✓	✓
n-Butyl Acetate	31272	BUTACET	123-86-4	$C_6H_{12}O_2$	116.2	CCCCOC(=O)C	✓	---
N-Methyl Formamide	31254	NMTF	123-39-7	C_2H_5NO	59.1	CNC=O	✓	✓
N-Methyl-2-Pyrrolidone	13387	NMP	872-50-4	C_5H_9NO	99.1	CN1CCCC1=O	✓	✓
Nitromethane	6375	NME	75-52-5	CH_3NO_2	61.0	C[N+](=O)[O-]	✓	✓
Octane	356	OCTE	111-65-9	C_8H_{18}	114.2	CCCCCCCC	✓	✓
Pentane	8003	PEN	109-66-0	C_5H_{12}	72.1	CCCCC	✓	✓
Pyridine	1049	PIR	110-86-1	C_5H_5N	79.1	C1=CC=NC=C1	✓	✓
t-Butyl Alcohol	6386	TBOH	75-65-0	$C_4H_{10}O$	74.1	CC(C)(C)O	✓	---
Tetrahydrofuran	8028	THF	109-99-9	C_4H_8O	72.1	C1CCOC1	✓	✓
Tetrahydropyran	8894	THP	142-68-7	$C_5H_{10}O$	86.1	C1CCOCC1	✓	✓
Toluene	1140	TOL	108-88-3	C_7H_8	92.1	CC1=CC=CC=C1	✓	✓
Water	962	WATER	7732-18-5	H_2O	18.0	O	✓	✓

Table S2. Crystallization experiments to identify new solvates and hydrates of CUR, DMC and BDMC.

Experiment number	Pure solvent* and solvent mixtures	Method
**1**	Pure solvent.	Heating to 50°C and slow evaporation to RT
**2**	Solvent and water	Heating to 50°C and slow evaporation to RT
**3**	Pure solvent.	Heating to 50°C and slow evaporation to 4 °C
**4**	Solvent and water	Heating to 50°C and slow evaporation to 4 °C
**5**	Pure solvent	Heating to 70°C and rapid cooling
**6**	Solvent and water	Heating to 70°C and rapid cooling
**7**	Solvent with water and acetone.	Heating to 50°C and slow evaporation to RT
**8**	Solvent with water and ethanol (CUR/BDMC) e Methanol (DMC)	Heating to 50°C and slow evaporation to RT
**9**	Solvent with water and dioxane.	Heating to 50°C and slow evaporation to RT
**10**	Pure solvent.	Heating to 50°C and rapid cooling
**11**	Pure solvent.	At 25 °C and Pressure 1.5 Bar
**12**	Pure solvent and ethanol-water mixture (10 - 90) v/v %	Heating to 50°C and slow evaporation to RT
**13**	Pure solvent	Heating to 30°C, rapid cooling in liquid nitrogen and slow evaporation at 4°C

* Pure solvent refers to the main solvent without any solvent **All samples were stirred at 1000 rpm using the BioShake thermoblock, model iQ.

Table S3. New solvate and hydrate forms for CUR, DCM and BDCM identified by co-crystallization experiments.

Polymorph I	Precipitated Solid	In solution	Not tested
**Pol I**	**p/p**	----	

**Solvent**	**CUR**												**BDMC**						**DMC**				
	**1**	**2**	**3**	**4**	**5**	**6**	**7**	**8**	**9**	**10**	**11**	**12**	**1**	**2**	**3**	**7**	**8**	**13**	**1**	**2**	**3**	**7**	**8**
DOX	----	**New solvate**	Pol I	----	----	Pol I				----	Pol I			p/p	p/p			**New solvate**		----			**New solvate**
BUT	Pol I	----	----	----	----	----			p/p				p/p									**p/p**	
6OH	----																						
8OH	----																						
5OH	P/P																						
PROH	Pol I	----	----	----	----	----	----	----	Pol I														
ISP	Pol I	Pol I							p/p				p/p		**Solvate**							**p/p**	
ACT	Pol I	----	----	Pol I	Pol I	Pol I	----	Pol I	p/p							----						**p/p**	
ACE	Pol I	Pol I							Pol I	----	Pol I	p/p	p/p						**p/p**			**p/p**	
ACN	Pol I	----					----	----	p/p	----	Pol I	p/p				Pol I				**New solvate**			----
ACP	----									----	----	p/p											
BZN	Pol I	Pol I					----	----	----							p/p						**p/p**	
CLB	Pol I	Pol I	----	----	----	----			P/P							----						**p/p**	
CLF	Pol I																						

Table S3 (continued).

Polymorph I **Pol I** | Precipitated Solid **p/p** | In solution **----** | Not tested

Solvent	CUR												BDMC						DMC				
	**1**	**2**	**3**	**4**	**5**	**6**	**7**	**8**	**9**	**10**	**11**	**12**	**1**	**2**	**3**	**7**	**8**	**13**	**1**	**2**	**3**	**7**	**8**
CLH	Pol I																						
CLP	----																						
CICLOPEN	----									----	----	p/p											
DET	Pol I	Pol I					p/p	p/p	----														
DMF	----	----	----	----	----	----				----	----	p/p											
DMSO	----	----	----	----	----	----	----	----	**New solvate**	----	----	p/p			p/p								----
EtOH	Pol I	Pol I							Pol I				p/p		p/p				----		----		
ETA	Pol I									----	Pol I	p/p											
HEP	----																						
HEX	Pol I	Pol I					Pol I		P/P													**p/p**	
DIE	----															---						**p/p**	
MCRES	----																						
MET	Pol I	Pol I							Pol I			p/p	**Solvate**			p/p	p/p		----	----	----		
MAC	Pol I									----	Pol I												
MEK	Pol I									----	Pol I	p/p											
DCM	Pol I									----	Pol I	p/p											
DMA	----									----	----												
NME	----	Pol I	Pol I	----	Pol I	----								p/p						----			
NMTF	----																						
NMP	----									----	----	p/p											
OCTE	----						Pol I																
PEN	----						----																
PIR	----									----	----												
THF	Pol I	Pol I					p/p	p/p	p/p	----	Pol I	p/p		p/p	p/p					----			
THP	p/p																						
TOL	Pol I	----	Pol I	Pol I	Pol I	Pol I			----							----						**p/p**	
WATER	----																	**New Hydrate**					

Table S4. Crystallographic information for crystal data and structure refinement of new solvates and hydrates for CUR, BDMC, and DMC.

Identification code	CUR-DOX	DMC-DOX	BDMC-DOX	BDMC-WATER1	BDMC-WATER2
Empirical formula	$C_{25}H_{28}O_8$	$C_{24}H_{26}O_7$	$C_{25}H_{28}O_7$	$C_{19}H_{18}O_5$	$C_{95}H_{90}O_{25}$
Formula weight	456.47	426.45	440.47	326.33	1631.66
Temperature/K	296 (2)	296 (2)	120 (2)	100	100
Crystal system	monoclinic	monoclinic	monoclinic	orthorhombic	monoclinic
Space group	$P2_1/n$	$P2_1$	Cc	Pcab	$P2_1/c$
a/Å	17.102(3)	11.7074(8)	26.377(4)	7.203(2)	20.255(5)
b/Å	6.9069(10)	5.6502(4)	5.7592(7)	10.789(1)	7.2390(6)
c/Å	20.123(3)	17.1885(12)	29.294(4)	41.120(4)	53.895(4)
α/°	90	90	90	90	90
β/°	95.213(8)	106.429(2)	90.871(6)	90	90.034(7)
γ/°	90	90	90	90	90
Volume/Å^3	2367.2(6)	1090.58(13)	4449.5(11)	3195.6(10)	7902(2)
Z	4	2	8	8	4
ρ_{calc}g/cm^3	1.281	1.299	1.315	1.357	1.371
μ/mm^{-1}	0.794	0.789	0.790	0.098	0.142
Radiation	CuKα (λ = 1.54178)	CuKα (λ = 1.54178)	CuKα (λ = 1.54178)	Synchrotron (λ = 0.71073)	Synchrotron (λ = 0.82656))
Reflections collected	33259	17236	20473	39909	207528
Independent reflections	4397 R_{int} = 0.0997 R_{sigma} = 0.0561	3438 R_{int} = 0.0586 R_{sigma} = 0.0440	5599 R_{int} = 0.1858 R_{sigma} = 0.1678	3814 R_{int} = 0.0428 R_{sigma} =0.0215	18872 R_{int} = 0.0764 R_{sigma} = 0.0267
Data/restraints/parameters	4397/264/358	3438/1/287	5599/956/638	3814/0/290	18872/0/1112
GOF on F^2	1.028	1.170	1.062	1.061	0.932
R (F) [I>=2σ (I)]	0.0676	0.0574	0.1176	0.0456	0.0434
wR (F^2) [I>=2σ (I)]	0.1766	0.1426	0.2794	0.1227	0.1358
R (F) [all data]	0.1158	0.0682	0.2156	0.0480	0.0664
wR (F^2) [all data]	0.2187	0.1488	0.3565	0.1262	0.1598

Table S5 List of all solvents tested with curcumin (**CUR**) to form solvates. Ranking and Consensus Ranking positions obtained through statistical (MC, CV, HBP) and thermodynamic (COSMO) methods.. The ✗ indicates that the solvent was tested, but the solvate form was not obtained (just precipitate). The ☐ indicates that the solvent was tested, resulting in the formation of polymorph I. The **---** indicates that the solvent was not tested. The ✓ indicates that the solvate was tested and the solvate form was obtained. New solvates obtained in this work and those reported in the literature are highlighted in bold green.

CUR	Ranking by techniques				Consensus ranking (CR)													
**Solvent**	**Cosmos**	**CV**	**MC**	**HBP**	**Cosmos-CV**	**Cosmos-MC**	**Cosmos-HBP**	**CV-MC**	**CV-HBP**	**HBP-MC**	**Cosmos-CV-MC**	**Cosmos-CV-HBP**	**CV-MC-HBP**	**MC-HBP-Cosmos**	**Cosmos-CV-MC-HBP**	**Tested by our team**	**Tested in literature**	**CSD Refcode**
**1,4-Dioxane**	**8**	**10**	**39**	**4**	**7**	**23**	**5**	**26**	**6**	**20**	**16**	**7**	**15**	**17**	**10**	✓	✓	**LADXEX / LADXIB**
1-Butanol	21	31	7	16	24	10	13	18	20	7	17	20	16	9	16	☐	---	-
1-Hexanol	26	35	5	17	35	12	20	19	26	6	23	28	18	14	20	✗	---	-
1-Octanol	35	36	1	14	39	16	27	17	22	1	26	34	14	16	23	✗	---	-
1-Pentanol	23	29	3	13	23	9	11	13	19	2	15	17	10	6	13	✗	---	-
1-Propanol	20	33	8	20	26	11	16	22	27	10	20	25	22	15	18	---	---	-
2-Methylfuran	37	21	11	32	33	25	37	12	28	21	24	35	23	30	30	---	---	-
2-Propanol	24	32	36	19	28	34	21	35	23	28	33	26	31	29	35	☐	☐	-
Acetic Acid	27	30	40	12	32	39	15	36	18	26	38	22	29	28	34	☐	---	-
**Acetone**	**10**	**11**	**30**	**7**	**9**	**20**	**8**	**20**	**10**	**15**	**13**	**9**	**13**	**12**	**9**	☐	✓	**FIHRUN**
Acetonitrile	18	17	24	18	17	21	12	21	13	19	18	11	19	19	17	☐	---	-
Acetophenone	17	14	4	27	13	7	22	2	17	12	5	14	9	13	12	✗	---	-
Benzene	38	-	38	-	-	42	-	-	-	-	44	-	-	44	36	☐	---	-
Chlorobenzene	39	23	19	37	36	28	39	23	37	30	29	39	26	38	39	☐	---	-
Chloroform	32	25	41	36	31	41	36	33	38	39	39	37	39	43	48	☐	---	-
Cyclohexane	45	-	42	-	-	47	-	-	-	-	47	-	-	47	45	☐	---	-
Cyclohexanone	41	3	18	9	20	29	28	3	4	9	21	12	4	23	14	---	---	-
Cyclopentane	43	-	44	-	-	46	-	-	-	-	48	-	-	48	46	✗	---	-
Cyclopentanone	12	1	27	6	6	19	9	9	3	14	8	4	6	10	6	✗	---	-
Diethyl Ether	14	19	2	38	14	1	30	6	31	18	6	23	21	18	15	☐	---	-
Dimethyl Formamide	4	2	35	3	1	18	2	16	1	17	10	2	7	7	5	✗	---	-
Dimethyl Sulfide	33	24	26	35	30	30	35	27	34	32	30	36	30	37	38	---	---	-
**Dimethyl Sulfoxide**	**1**	**5**	**17**	**1**	**2**	**4**	**1**	**7**	**2**	**3**	**1**	**1**	**1**	**1**	**1**	✓	---	-
Ethanol	19	28	33	21	22	27	17	31	21	27	28	21	28	25	29	☐	☐	-
Ethyl Acetate	29	13	21	25	19	26	32	14	15	23	22	19	20	26	24	☐	---	-
Ethylene	36	-	43	-	-	45	-	-	-	-	46	-	-	45	40	---	---	-
Formic Acid	31	22	46	29	25	43	33	34	24	37	40	31	36	41	44	---	---	-
Heptane	47	-	13	-	-	32	-	-	-	-	32	-	-	34	26	✗	---	-
Hexane	46	-	16	-	-	35	-	-	-	-	36	-	-	36	27	☐	---	-
Isopropyl Ether	13	20	10	39	15	8	31	10	35	24	11	24	24	20	19	✗	---	-
m-Cresol	16	38	22	28	27	17	23	30	39	25	27	30	32	21	32	✗	---	-
Methanol	22	34	45	23	29	38	25	38	30	35	42	29	37	32	42	☐	☐	-
**Methyl Acetate**	**28**	**12**	**20**	**24**	**18**	**24**	**29**	**11**	**14**	**22**	**19**	**16**	**17**	**24**	**21**	☐	✓	-
Methyl Ethyl Ketone	11	9	9	11	8	5	10	1	11	5	4	10	3	4	4	☐	---	-
**Methylene Dichloride**	**34**	**26**	**32**	**34**	**34**	**36**	**34**	**29**	**36**	**34**	**34**	**38**	**33**	**39**	**43**	☐	✓	**OJIWOV**
N,N-Dimethyl Acetamide	2	8	15	5	4	2	3	8	5	4	3	3	2	2	2	✗	---	-
n-Butyl Acetate	15	15	6	26	12	6	18	5	16	13	7	13	12	11	11	---	---	-
Nitromethane	42	4	47	30	21	48	38	28	12	38	35	27	27	46	41	☐	---	-
N-Methyl Formamide	7	27	37	31	16	22	14	32	32	36	25	18	35	27	31	☐	---	-
N-Methyl-2-Pyrrolidone	3	7	14	10	3	3	7	4	8	8	2	6	5	3	3	✗	---	-
Octane	48	-	12	-	-	33	-	-	-	-	31	-	-	33	25	☐	---	-
Pentane	44	-	23	-	-	37	-	-	-	-	41	-	-	40	28	✗	---	-
Pyridine	5	6	29	8	5	13	6	15	7	16	9	5	8	8	7	✗	---	-
t-Butyl Alcohol	25	37	34	22	37	31	26	37	33	29	37	32	34	31	37	---	---	-
Tetrahydrofuran	6	16	28	2	10	14	4	25	9	11	12	8	11	5	8	☐	---	-
Tetrahydropyran	9	18	25	33	11	15	19	24	25	31	14	15	25	22	22	✗	---	-
Toluene	40	-	31	-	-	40	-	-	-	-	43	-	-	42	33	☐	---	-
**Water**	**30**	**39**	**48**	**15**	**38**	**44**	**24**	**39**	**29**	**33**	**45**	**33**	**38**	**35**	**47**	✗	✓	-

Table S6 List of all solvents tested with bisdemethoxycurcumin (**BDMC**) to form solvates. Ranking and Consensus Ranking positions obtained through statistical (MC, CV, HBP) and thermodynamic (COSMO) methods.. The ✗ indicates that the solvent was tested, but the solvate form was not obtained (just precipitate). The ☐ indicates that the solvent was tested, resulting in the formation of polymorph I. The **---** indicates that the solvent was not tested. The ✓ indicates that the solvate was tested and the solvate form was obtained. New solvates obtained in this work and those reported in the literature are highlighted in bold green.

**BDMC**	**Ranking by techniques**				**Consensus ranking (CR)**													
**Solvent**	**Cosmos**	**CV**	**MC**	**HBP**	**Cosmos-CV**	**Cosmos-MC**	**Cosmos-HBP**	**CV-MC**	**CV-HBP**	**HBP-MC**	**Cosmos-CV-MC**	**Cosmos-CV-HBP**	**CV-MC-HBP**	**MC-HBP-Cosmos**	**Cosmos-CV-MC-HBP**	**Tested by our team**	**Tested in literature**	**CSD Refcode**
**1,4-Dioxane**	**8**	**9**	**35**	**4**	**7**	**20**	**6**	**24**	**6**	**19**	**14**	**7**	**11**	**11**	**9**	✓	✓	**-**
1-Butanol	21	31	9	29	24	12	25	22	31	18	20	26	24	19	26	✗	---	-
1-Hexanol	24	35	2	28	32	10	30	17	37	12	19	29	23	15	25	---	---	-
1-Octanol	29	36	1	23	37	13	31	18	30	4	23	30	19	14	24	---	✗	-
1-Pentanol	22	29	3	26	23	8	21	13	26	10	15	24	16	13	19	---	---	-
1-Propanol	20	33	24	30	25	22	26	28	38	29	28	28	31	27	34	---	---	-
2-Methylfuran	37	21	7	31	31	23	34	10	24	17	22	34	18	28	30	---	---	-
**2-Propanol**	**25**	**32**	**38**	**14**	**28**	**34**	**17**	**35**	**20**	**25**	**37**	**22**	**29**	**29**	**35**	✓	✓	**WUWLUY / XIWDEP**
Acetic Acid	27	30	43	24	30	41	29	36	25	35	41	27	35	36	41	✗	✗	-
**Acetone**	**9**	**10**	**30**	**11**	**8**	**19**	**9**	**21**	**9**	**21**	**13**	**9**	**13**	**12**	**12**	✓	✓	**XIWDAL**
Acetonitrile	17	18	40	13	18	30	13	29	15	26	27	14	25	25	23	☐	✓	-
Acetophenone	18	13	6	19	14	6	15	2	16	5	5	16	8	8	8	---	---	-
Benzene	38	-	27	-	-	37	-	-	-	-	40	-	-	41	32	✗	---	-
Chlorobenzene	40	23	10	39	36	26	39	14	35	24	26	39	26	33	36	✗	---	-
Chloroform	35	25	36	38	34	42	37	31	39	37	38	38	36	43	47	---	---	-
Cyclohexane	45	-	37	-	-	46	-	-	-	-	47	-	-	47	40	---	---	-
Cyclohexanone	42	4	13	7	20	28	22	1	4	1	18	17	1	22	13	---	---	-
Cyclopentane	43	-	44	-	-	47	-	-	-	-	48	-	-	48	45	---	---	-
Cyclopentanone	12	3	23	6	6	15	8	6	3	9	7	6	4	7	6	---	---	-
Diethyl Ether	16	16	5	34	15	1	24	3	22	20	6	19	15	16	14	---	---	-
Dimethyl Formamide	4	2	33	2	2	17	3	15	2	15	8	2	6	6	5	---	---	-
Dimethyl Sulfide	32	24	29	36	27	32	35	27	33	32	31	36	32	39	39	---	---	-
**Dimethyl Sulfoxide**	**1**	**1**	**25**	**1**	**1**	**9**	**1**	**5**	**1**	**6**	**1**	**1**	**2**	**2**	**1**	✗	✓	**-**
Ethanol	19	28	34	21	22	27	20	32	21	30	29	20	28	26	33	✗	☐	-
Ethyl Acetate	28	15	21	12	19	25	19	16	11	14	21	18	12	21	17	---	---	-
Ethylene	36	-	42	-	-	44	-	-	-	-	45	-	-	45	37	---	---	-
Formic Acid	33	22	45	33	26	43	33	34	27	38	42	31	37	44	46	---	---	-
Heptane	47	-	17	-	-	36	-	-	-	-	39	-	-	38	31	---	---	-
Hexane	46	-	11	-	-	31	-	-	-	-	32	-	-	32	21	✗	---	-
Isopropyl Ether	14	20	8	37	17	3	27	9	28	22	11	21	22	18	18	✗	---	-
m-Cresol	30	38	4	22	38	14	32	23	32	7	24	35	20	17	28	---	---	-
**Methanol**	**23**	**34**	**46**	**16**	**29**	**40**	**18**	**38**	**23**	**31**	**43**	**23**	**34**	**31**	**38**	✓	✓	**BUWKUZ / WUWLOS**
Methyl Acetate	15	11	28	20	11	21	14	20	14	23	16	12	17	23	15	---	✗	-
Methyl Ethyl Ketone	11	19	12	10	13	4	10	12	12	2	10	10	9	5	7	---	--	-
Methylene Dichloride	34	26	32	35	33	38	36	30	34	36	33	37	33	42	43	---	--	-
N,N-Dimethyl Acetamide	2	8	20	3	3	2	2	7	5	3	2	3	3	1	2	---	--	-
n-Butyl Acetate	13	14	14	17	12	11	12	8	13	13	9	11	10	10	10	---	✗	-
Nitromethane	41	5	47	32	21	48	38	26	17	39	34	25	30	46	42	✗	---	-
N-Methyl Formamide	6	27	39	15	16	24	11	33	19	28	25	13	27	20	22	---	---	-
N-Methyl-2-Pyrrolidone	3	7	22	8	4	7	4	11	8	11	4	4	7	4	4	---	---	-
Octane	48	-	15	-	-	35	-	-	-	-	36	-	-	37	29	---	---	-
Pentane	44	-	18	-	-	33	-	-	-	-	35	-	-	35	27	---	---	-
Pyridine	5	6	19	9	5	5	7	4	7	8	3	5	5	3	3	---	---	-
t-Butyl Alcohol	26	37	41	25	35	39	28	37	36	34	44	32	38	34	44	---	---	-
**Tetrahydrofuran**	**7**	**17**	**31**	**5**	**10**	**18**	**5**	**25**	**10**	**16**	**17**	**8**	**14**	**9**	**11**	✗	✓	**-**
Tetrahydropyran	10	12	26	27	9	16	16	19	18	27	12	15	21	24	16	---	---	-
Toluene	39	-	16	-	-	29	-	-	-	-	30	-	-	30	20	✗	---	-
**Water**	**31**	**39**	**48**	**18**	**39**	**45**	**23**	**48**	**29**	**33**	**46**	**33**	**39**	**40**	**48**	✓	✓	**GANJAG**

…….

Table S7 List of all solvents tested with demethoxycurcumin (**DMC)** to form solvates. Ranking and Consensus Ranking positions obtained through statistical (MC, CV, HBP) and thermodynamic (COSMO) methods.. The ✗ indicates that the solvent was tested, but the solvate form was not obtained (just precipitate). The ☐ indicates that the solvent was tested, resulting in the formation of polymorph I. The **---** indicates that the solvent was not tested. The ✓ indicates that the solvate was tested and the solvate form was obtained. New solvates obtained in this work and those reported in the literature are highlighted in bold green.

**DMC**	**Ranking by techniques**			**Consensus ranking (CR)**				**Tested by our team**	**Tested in literature**	**CSD Refcode**
**Solvent**	**Cosmos**	**CV**	**HBP**	**Cosmos-CV**	**Cosmos-HBP**	**CV HBP**	**Cosmos CV HBP**			
**1,4-Dioxane**	**8**	**10**	**7**	**7**	**7**	**8**	**6**	✓	---	---
1-Butanol	22	31	20	24	24	25	25	✗	---	---
1-Hexanol	25	35	17	34	34	26	28	---	---	---
1-Octanol	30	36	18	38	38	30	31	---	---	---
1-Pentanol	23	29	19	23	23	21	23	---	---	---
1-Propanol	21	33	21	25	25	29	26	---	---	---
2-Methylfuran	37	21	38	32	32	34	36	---	---	---
2-Propanol	26	32	26	31	31	31	32	✗	---	---
Acetic Acid	28	30	8	29	29	16	21	✗	---	---
Acetone	10	13	6	10	10	9	8	✗	---	---
**Acetonitrile**	**18**	**12**	**13**	**12**	**12**	**12**	**11**	✓	---	---
Acetophenone	17	16	27	15	15	18	17	---	---	---
Benzene	38	-	-	-	-	-	-	✗	---	---
Chlorobenzene	40	23	35	36	36	32	38	✗	---	---
Chloroform	34	25	39	33	33	38	39	---	---	---
Cyclohexane	45	-	-	-	-	-	-	---	---	---
Cyclohexanone	41	4	9	20	20	6	13	---	---	---
Cyclopentane	43	-	-	-	-	-	-	---	---	---
Cyclopentanone	12	3	11	6	6	7	7	---	---	---
Diethyl Ether	15	19	31	18	18	24	19	---	---	---
Dimethyl Formamide	4	2	2	2	2	2	2	---	---	---
Dimethyl Sulfide	32	24	37	27	27	35	35	---	---	---
Dimethyl Sulfoxide	1	1	1	1	1	1	1	✗	---	---
Ethanol	20	28	15	22	22	19	18	✗	---	---
Ethyl Acetate	29	14	10	19	19	11	12	---	---	---
Ethylene	36	-	-	-	-	-	-	---	---	---
Formic Acid	33	22	24	26	26	20	29	---	---	---
Heptane	47	-	-	-	-	-	-	---	---	---
Hexane	46	-	-	-	-	-	-	✗	---	---
Isopropyl Ether	13	20	34	17	17	28	22	✗	---	---
m-Cresol	19	38	28	28	28	39	33	---	---	---
Methanol	24	34	14	30	30	22	24	---	---	---
Methyl Acetate	16	15	23	13	13	15	14	---	---	---
Methyl Ethyl Ketone	11	9	3	9	9	4	5	---	---	---
Methylene Dichloride	35	26	36	35	35	37	37	---	---	---
N,N-Dimethyl Acetamide	2	8	5	3	3	5	4	---	---	---
n-Butyl Acetate	14	18	25	14	14	17	15	---	---	---
Nitromethane	42	5	29	21	21	13	27	✗	---	---
N-Methyl Formamide	6	27	32	16	16	33	20	---	---	---
N-Methyl-2-Pyrrolidone	3	7	30	4	4	14	10	---	---	---
Octane	48	-	-	-	-	-	-	---	---	---
Pentane	44	-	-	-	-	-	-	---	---	---
Pyridine	5	6	4	5	5	3	3	---	---	---
t-Butyl Alcohol	27	37	16	37	37	27	30	---	---	---
Tetrahydrofuran	7	11	12	8	8	10	9	✗	---	---
Tetrahydropyran	9	17	33	11	11	23	16	---	---	---
Toluene	39	-	-	-	-	-	-	✗	---	---
Water	31	39	22	39	39	36	34	✗	---	---

Table S8 Comparison of the ranking and consensus ranking of prediction methods for solvates.

	CUR		BDMC	
	**Prediction Methods**	$\frac{\sum_{i=1}^{n} P_{m,i}}{6}$	**Prediction Methods**	$\frac{\sum_{i=1}^{n} P_{m,i}}{7}$
**Ranking by techniques**	HBP	**14.2**	HBP	**9.9**
	CV	**17.2**	Cosmos	**14.9**
	Cosmos	**18.5**	CV	**20.3**
	MC	**31.0**	MC	**36.1**
**Consensus Ranking**	CV-HBP	**16.2**	Cosmos-HBP	**11.3**
	Cosmos-HBP	**16.8**	CV-HBP	**14.0**
	Cosmos-CV-HBP	**17.3**	Cosmos-CV-HBP	**14.7**
	Cosmos-CV	**18.0**	Cosmos-CV	**17.4**
	CV-MC-HBP	**19.5**	MC-HBP-Cosmos	**19.1**
	HBP-MC	**21.2**	CV-MC-HBP	**20.3**
	Cosmos-CV-MC	**21.3**	HBP-MC	**21.6**
	MC-HBP-Cosmos	**21.3**	Cosmos-CV-MC-HBP	**22.0**
	Cosmos-CV-MC-HBP	**21.8**	Cosmos-CV-MC	**24.4**
	CV-MC	**22.0**	Cosmos-MC	**26.4**
	Cosmos-MC	**25.2**	CV-MC	**28.0**

Worked-out example

This section illustrates a worked example for the calculation of ΔCV for each component: the target molecule (IFA), the solvent, and the multicomponent system (IFA–solvent).

The coordination values for each structure were obtained using Python scripts based on the CSD Python API. From these calculations, the donor (D) and acceptor (A) capacities were derived for the isolated molecules (API and solvent) and for the multicomponent form (solvate), as shown below:

	**Identifier**	**D_sum**	**A_sum**
**IFA**	CUR	1.977	1.361
**Solvent**	1,4 Dioxane	0.000	0.427
**Multicomponent**	Solvate (CUR-1,4 dioxane)	2.096	1.412

Next, the Equation 3 was applied to calculate ΔCV:

$$\Delta CV = (|(D - A)_{\text{solvate}}| - |(D - A)_{\text{target molecule}}|) + (|(D - A)_{\text{solvate}}| - |(D - A)_{\text{solvent}}|) \qquad \textbf{Eq 3}$$

Substituting the numerical values:

$$\Delta CV = (|(2.096 - 1.412)_{\text{solvate}}| - |(1.977 - 1.361)_{\text{target molecule}}|) + (|(2.096 - 1.412)_{\text{solvate}}| - |(0.000 - 0.427)_{\text{solvent}}|)$$

$$\Delta CV = (|0.684| - |0.616|) + (|0.684| - |-0.427|)$$

$$\Delta CV = (0.684 - 0.616) + (0.684 - 0.427)$$

$$\Delta CV = 0.068 + 0.257$$

$$\Delta CV = \mathbf{0.325}$$

Thus, the calculated ΔCV for the CUR–1,4-dioxane is **0.325**.
